# Data on cities that are benchmarked with the sustainable development of energy, water and environment systems index and related cross-sectoral scenario

**DOI:** 10.1016/j.dib.2019.103856

**Published:** 2019-03-20

**Authors:** Şiir Kılkış

**Affiliations:** The Scientific and Technological Research Council of Turkey (TÜBİTAK), Atatürk Bulvarı No: 221, Kavaklıdere 06100 Ankara, Turkey

**Keywords:** Energy, Water, Environment, Sustainable development, Cities, Composite indicator

## Abstract

The data set of this article is related to an original research article entitled “Benchmarking the sustainability of urban energy, water and environment systems and envisioning a cross-sectoral scenario for the future” Kılkış, 2019. The data article provides data compilations in the context of benchmarking studies based on the composite indicator of the Sustainable Development of Energy, Water and Environment Systems City Index. Data tables for the seven dimensions of the index are provided for 35 main indicators and related sub-indicators for the newly benchmarked cities while those for other cities are monitored. In addition to periodic updates in the common data sources, some cities released updated reports for the Sustainable Energy and/or Climate Action Plans and/or relevant local statistics since the initial benchmarking. Normalized and aggregated values per dimension of the index for 120 cities are provided as an appendix for groups of 30 cities that are characterized as the pioneering, transitioning, solution-seeking, and challenged cities of the sample. The data compilation for the sources of residual energy from the industry, thermal power generation, the wastewater sector and urban biowaste are further provided for 60 cities as the basis of a scenario to encourage the integration of cross-sectoral measures in urban systems to improve benchmarked performances. The data that is contained in this data article thus enables the original application of the index to 120 cities and the analysis of a scenario in which cities reduce primary energy spending and carbon dioxide emissions.

**Table:** Specifications Table

Subject area	*Energy*
More specific subject area	*Renewable Energy, Sustainability and the Environment*
Type of data	*Tables and Figure*
How data was acquired	*Compiled based on a comprehensive horizon-scanning of data sources that are processed for a composite indicator and scenario.*
Data format	*Formatted data (*Table 1, Table 2, Table 3, Table 4, Table 5, Table 6, Table 7, Table 8, Table 9, Table 10*and*Tables A1-A10*in*Appendix A*); Processed and analyzed data (*Fig. 1*,*Appendix BTables B1-B4*).*
Experimental factors	*Cities are selected for benchmarking based on criteria for data availability, geographical diversity and researcher representation in the scientific platform in which the index results are shared.*
Experimental features	*Compiled and formatted data compilations are utilized for data processing and analyses in the context of the research work.*
Data source location	*Cities around the world, including Aalborg, Birmingham, Bologna, Cape Town, Christchurch, Constanţa, Dublin, Funchal, Gdynia, Glasgow, Hamburg, Johannesburg, Murcia, Reykjavík, Riga, Sfax, Sydney and Tallinn among a total of 120 cities. Data on residual energy for 60 cities are compiled for a cross-sectoral scenario.*
Data accessibility	*The data article contains*Table 1, Table 2, Table 3, Table 4, Table 5, Table 6, Table 7, Table 8, Table 9, Table 10*and*Fig. 1*. Supplementary data sets that are associated with this data article based on*Appendix A*for*Tables A1-A10*and*Appendix B*for*Tables B1-B4*can be found in the online version, which is available at*https://doi.org/10.1016/j.dib.2019.103856Appendix C*that contains additional references is similarly accessible. The atlas depicted in*Fig. 1*is accessible at*http://www.sdewes.org/sdewes_index.php*.*
Related research article	*Ş. Kılkış, Benchmarking the sustainability of urban energy, water and environment systems and envisioning a cross-sectoral scenario for the future, Renew Sust Energ Rev 103 (2019) 529–545.*https://doi.org/10.1016/j.rser.2018.11.006*[1]**.*

**Table:** 

**Value of the data** • The data tables are of value to the scientific community based on the provision of original data compilations for cities, which can be used within and beyond the context of the Sustainable Development of Energy, Water and Environment Systems City Index. • The data can be used to facilitate comparisons with future results and measure progress towards more sustainable urban energy, water and environment systems across time. • The data can be used to devise additional scenarios for cities beyond the scenario that is considered in the related research article for utilizing residual heat and urban biowaste. • The data provides a basis for new research undertakings that are directed to improving the existing performance of cities and city collaboration pairs while serving as a benchmark to compare, envision and realize more sustainable urban systems in the future.

## 1 Data

This article contains data compilations for the 35 main indicators in the seven dimensions of the Sustainable Development of Energy, Water and Environment Systems (SDEWES) City Index for 18 newly benchmarked cities. These data compilations are provided in data tables for the main indicators of each dimension in this article while data inputs for the sub-indicators are provided in data tables in Appendix A. As the companion data article of an original research article [1] that provides a benchmarking study for 120 sampled cities and the application of a cross-sectoral scenario, other tables contain updated data sources for 25 cities in comparison to references [2], [3], [4], [5], [6] and data on the theoretically available sources of residual energy in the urban vicinity for 60 cities. The latter includes data on the residual heat from industry, thermal power generation, the wastewater sector as well as urban biowaste based on city level data compilations using the Pan-European Thermal Atlas (Peta) [7] and related local maps of the STRATEGO project [8].

**Fig. 1:**
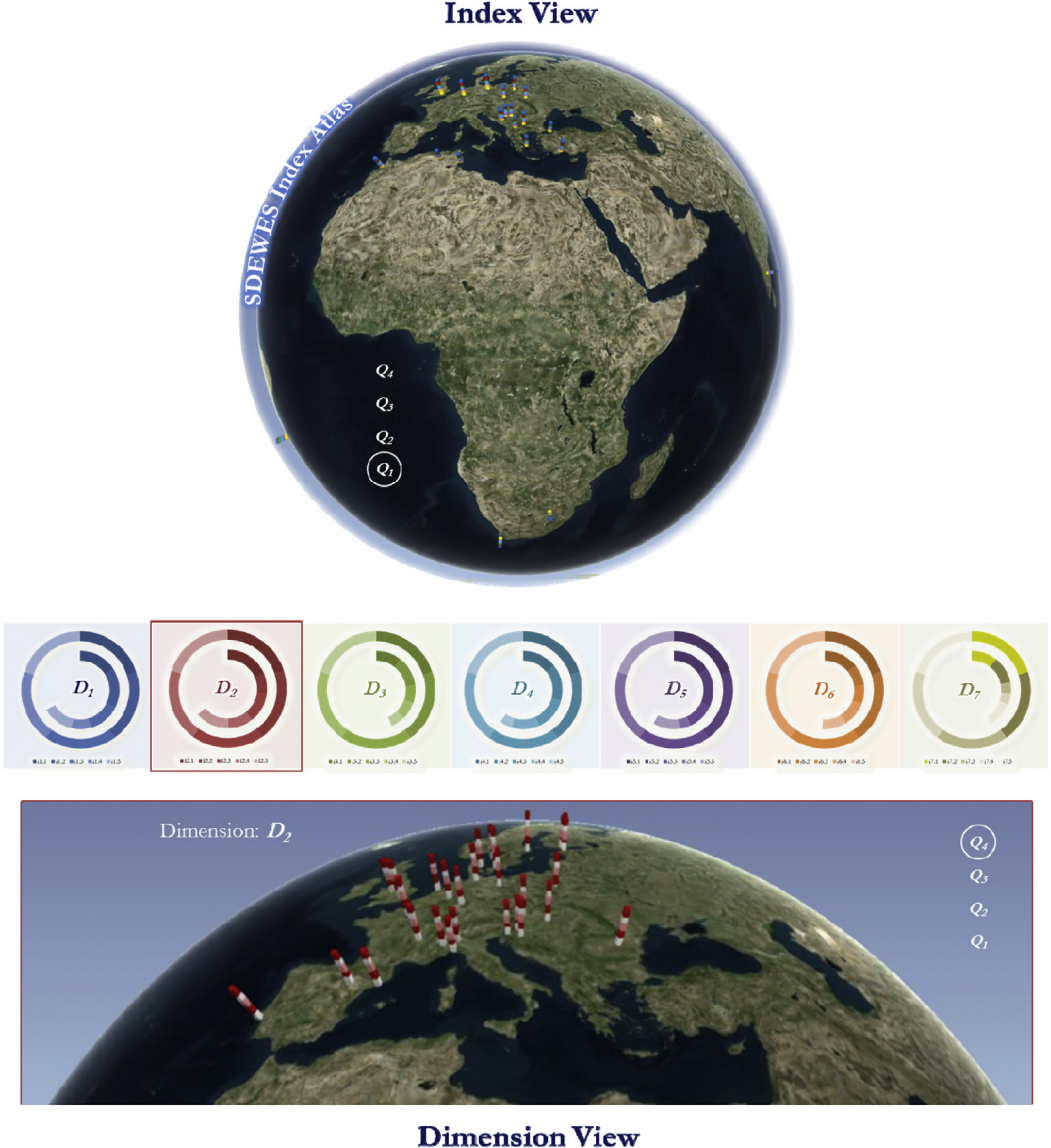
Processing of the compiled and analyzed data sets for a SDEWES index atlas.

In the context of processed and analyzed data, Fig. 1 in this article represents the layers of an atlas of the index results and Appendix B includes the data set for the normalized and aggregated values of 120 benchmarked cities per each dimension of the index. The data set of Appendix B is organized into four tables according to the top 30 cities that are characterized as the pioneering cities, the cities that are ranked 31–60 as the transitioning cities and the cities that are ranked 61–120, which contain two groups for the solution-seeking and challenged cities, respectively. These tables also correspond to the organization of the layers in the atlas that is represented in Fig. 1.

Overall, 10 tables are provided in the data article and 14 tables are provided in Appendices A and B for a total of 24 tables, which provide the basis of the research work in reference [1] for a comprehensive benchmarking of 120 cities. Additional references are provided in Appendix C. Beyond the present context, the data is relevant for Sustainable Development Goals 6, 7 and 11 on clean energy, water and sustainable cities and communities among others [9], the Global Covenant of Mayors for Climate & Energy Initiative [10] and a comprehensive assessment of urban progress for various studies, including the Sixth Assessment Report of the Intergovernmental Panel on Climate Change [11].

## 2 Experimental design, materials and methods

According to the scope of the data article, this section provides the means of acquiring data for seven dimensions of the index *D*_*1*_ to *D*_*7*_ to perform analyses for 120 cities. Table 1 puts forth the 18 newly benchmarked cities in alphabetical order from Aalborg to Tallinn along with the main strategic references [12–53]^1^ some of which are based on the Covenant of Mayors (CoM) initiative [54] and Climate Leadership Group (C40) [55]. As indicated in the specifications table, the benchmarked cities are selected based on criteria for data availability (*M*_*1*_), geographical diversity (*M*_*2*_), and representation in the scientific platform in which the index results are shared (*M*_*3*_). Cities from a particular country are prioritized according to population in descending order. This is necessary given that CoM signatories can have populations less than 10,000 inhabitants.

**Table 1:** Summary of the 18 newly benchmarked cities in the sample of 120 cities.

City (*C*_*j*_)	Country	*M*_*1*_^a^	*M*_*2*_	*M*_*3*_	Reference	Initiative
Aalborg	Denmark	8	2	++	[12–15]	CoM
Birmingham	UK/England	2	1	++	[16, 17]	CoM
Bologna	Italy	292	9	++++	[18, 19]	CoM
Cape Town	South Africa	18^b^	0	+	[20–22]	C40
Christchurch	New Zealand	1	0	+	[23, 24]	CoM
Constanța	Romania	4	4	+	[25]	CoM
Dublin	Ireland	2	0	+	[26, 27]	CoM
Funchal	Portugal	20	4	+	[28–30]	CoM
Gdynia	Poland	3	2	++	[31, 32]	CoM
Glasgow	UK/Scotland	2	0	++	[33–35]	CoM
Hamburg	Germany	7	3	+++	[36–38]	CoM
Johannesburg	South Africa	18^b^	0	+	[20]	C40
Murcia	Spain	174	7	++	[39, 40]	CoM
Reykjavík	Iceland	1	0	–	[41–44]	CoM
Riga	Latvia	4	0	+	[45]	CoM
Sfax	Tunisia	1	0	+	[46]	CoM
Sydney	Australia	2 (19)^c^	0	+	[47–50]	C40
Tallinn	Estonia	1	0	+	[51, 52]	CoM

a The number of monitoring reports in 2017 or recent SEAP/SECAP plans [54] unless specified otherwise.

b The number of municipalities with local level data rather than CoM signatories for geographical diversity.

c Represents C40 cities and major cities in the State of Australian Cities [53] with limited energy data.

The process of acquiring data is facilitated by the involvement of cities in climate initiatives. One data source is updates from CoM signatories based on the progress of completed and ongoing actions at least every 2 years and monitoring inventories every 4 years [56]. Data sources for local statistics may be available annually if reported in the same scope. In addition to the cities in Table 1, a dedicated questionnaire^2^ was sent to the contact points of the 102 benchmarked cities to confirm the availability of any local reporting in addition to any updated monitoring reports on the CoM website [57]. The contact points were managers for urban energy and environment issues and those responsible for the monitoring of Sustainable Energy Action Plans (SEAP), Sustainable Energy and Climate Action Plans (SECAP) and/or other equivalent local plans and strategies. Such aspects addressed related data challenges in the process of pursuing data inputs.

Updated data sources for 25 cities based on [58–94] were identified, including new monitoring reports as retrieved from the CoM database [57]. The energy and sustainability managers of cities also provided additional resources for local statistics, including those for Espoo in a Finnish emissions database at the local level [75]. Table 2 identifies the cities with updated references for climate mitigation related plans and statistics since the initial benchmarking of a city in references [2], [3], [4], [5], [6]. Other updates in the data sources include those based on a newer version of the World Health Organization (WHO) Global Urban Ambient Air Pollution Database [95] that provides a basis to compile data inputs for the annual mean particulate matter concentrations less than 10 μm in diameter (PM_10_) based on urban monitoring station readings. All other data sources, including the Urban Waste Water Treatment Directive (UWWTD) database [96, 97], were comprehensively re-assessed and reviewed to determine any changes for the 120 cities.

**Table 2:** Cities with updated monitoring data since initial benchmarking studies [2], [3], [4], [5], [6].

Initial Benchmarking^a^	City (*C*_*j*_)	Updated Monitoring Source^b^, ^c^
SEE cities [2], [5]	Athens	[58]
	Belgrade	[59]
	Ohrid	[60]
	Zagreb	[61]
	Budapest	[62]
Mediterranean port cities [3]	Barcelona	[63]
	Málaga	[64]
	Rijeka	[65]
	Dubrovnik	[66, 67]
World cities [4]	Århus	[68–71]
	Bogotá	[72]
	Cologne	[73]
	Eskişehir Tepebaşı	[74]
	Espoo	[75]
	Frankfurt	[76]
	Lisbon	[77]
	Milan	[78, 79]
	Nagoya	[80]
	Paris	[81–83]
	Pisa	[84]
	Stockholm	[85–88]
	Vienna	[89]
	Warsaw	[90]
	Washington D.C.	[91, 92]
	Zaragoza	[93]

a Excluding the cities benchmarked in [6] for which updated monitoring data is found to be already integrated.

b Also includes references that were shared by the city energy managers through the index questionnaire.

c In some cities, such as Zadar, the recent monitoring report was only for actions rather than data [94].

The data article proceeds with a dimension by dimension description of the original data acquisition process for 18 newly benchmarked cities while the original data compilation for 120 cities are represented based on the average (mean) value in the sample that is marked for *C*_*AV*_. The normalized and aggregated values per dimension for 120 cities in the sample are appended.

### 2.1 Data compilation on energy usage and climate

Table 3 represents the process of acquiring data for the first dimension on “Energy Usage and Climate” (*D*_*1*_) based on data inputs for the urban energy system. The main data sources are sufficient to attain data inputs on energy usage in the building sector for residential, tertiary, and municipal buildings and the transport sector based on the energy usage of private, public, and municipal vehicle fleets. The total energy usage of buildings, transport, industry (non-ETS) and public lighting is evaluated on a per capita basis and calculated for each city when necessary. Other aspects of energy usage include climate and the overall efficiency with which primary energy spending is used, including energy production, transmission, and distribution. In this respect, a total degree days factor and the final to primary energy ratio are obtained for the data compilation.

**Table 3:** Data inputs to the energy usage and climate dimension (*D*_*1*_).

Indicators per City (*C*_*j*_)	*i*_1.1_	*i*_1.2_	*i*_1.3_	*i*_1.4_	*i*_1.5_
	Energy usage of buildings (MWh)	Energy usage of transport (MWh)	Energy usage per capita (MWh/capita)	Total degree days factor^b^	Final to primary energy ratio (%)
Data Sources	SEAP^a^	SEAP^a^	SEAP^a^	[98]	[99]
Aalborg	5,542,778	1,072,958	31.84	1158	80
Birmingham	12,826,000	4,369,000	15.67	999	69
Bologna	5,245,000	1,441,075	19.58	1086	78
Cape Town	11,278,180	28,052,404	11.23	980	54
Christchurch	2,746,000	7,126,000	32.80	862	69
Constanţa	1,295,527	352,632	6.97	1159	72
Dublin	6,964,227	2,741,604	19.22	958	79
Funchal	524,800	577,793	10.44	977	76
Gdynia	1,952,000	351,000	12.11	1116	69
Glasgow	8,146,400	3,053,200	18.30	1025	69
Hamburg	22,884,444	13,217,222	24.79	1085	72
Johannesburg	7,513,655	30,901,963	9.92	961	54
Murcia	1,777,000	2,551,000	9.93	1058	71
Reykjavík	3,161,455	932,376	10.26	1392	47
Riga	7,478,769	2,942,467	18.87	1311	89
Sfax	563,113	1,197,018	8.40	1248	69
Sydney	7,981,445	1,315,504	9.30	989	64
Tallinn	3,916,000	3,372,000	20.49	1370	49
*Average (18 cities)*	*6,210,933*	*5,864,845*	*16.12*	*1096*	*68*
*Average City* (*C*_*AV*_)	*7,976,131*	*4,649,456*	*14.89*	*1145*	*71*

a Obtained or calculated from SEAP or equivalent plans based on the references in Table 1 [12–52].

b Weighted by an average COP of 4 in the heating season and an average COP of 3.5 in the cooling season.

### 2.2 Data compilation on penetration of energy and carbon dioxide saving measures

The means of acquiring data for dimension *D*_*2*_ on “Penetration of Energy and CO_2_ Saving Measures” necessitates an evaluation of the strategic actions of the city for climate mitigation. Table 4 represents the data inputs for the main indicators of *D*_*2*_ while those for the sub-indicators are provided in Appendix A. In this appendix, Table A1 represents the data acquisition process for evaluating the energy system characteristics considering combined heat and power (CHP) based on district heating and/or cooling (DH/C) networks, the use of renewable energy sources, including geothermal energy, and progress towards a climate neutral heating sector in 18 cities. Table A2 represents the data acquisition process for evaluating the implementation status of nearly net-zero energy buildings and/or districts. Other aspects of data acquisition for *D*_*2*_ require a comprehensive evaluation for public transport, including bus, trolleybus, and/or urban rail options for the transport sector (Table A3). These data inputs include urban rail density and daily ridership as well as the use of such decentralized options as bicycle sharing in support of the public transport network. Numerous local sources are used to acquire such data inputs in *D*_*2*_ for each city, including those for cities that have higher levels of penetration in solid-state lighting and solar energy based armatures.

**Table 4:** Data inputs to the penetration of energy and CO_2_ measures dimension (*D*_*2*_).

Indicators per City (*C*_*j*_)	*i*_2.1_	*i*_2.2_	*i*_2.3_	*i*_2.4_	*i*_2.5_
	Action Plan for Energy and CO_2_ Emissions^a^	Combined heat and power based DH/C	Energy savings in end-usage (buildings)	Density of public transport network	Efficient public lighting armatures^e^
Data Sources	[12–52]	Table A1^b^	Table A2^c^	Table A3^d^	[12–52]
Aalborg	2.0	2.0	2.0	2.0	1.0
Birmingham	2.0	2.0	2.0	2.5	2.0
Bologna	2.0	2.0	2.0	2.0	1.0
Cape Town	1.0	0.0	1.0	1.5	1.0
Christchurch	2.0	1.0	2.0	2.0	1.0
Constanţa	2.0	1.0	1.0	1.5	1.0
Dublin	2.0	1.0	2.0	3.0	1.0
Funchal	2.0	0.0	1.0	1.0	1.0
Gdynia	2.0	1.0	1.0	1.0	1.0
Glasgow	2.0	1.0	2.0	3.0	2.0
Hamburg	2.0	2.0	2.0	4.0	2.0
Johannesburg	1.0	0.0	1.0	1.0	1.0
Murcia	2.0	2.0	1.0	1.5	1.0
Reykjavík	2.0	3.0	1.0	1.5	2.0
Riga	2.0	2.0	1.0	3.0	2.0
Sfax	2.0	0.0	1.0	1.0	1.0
Sydney	2.0	1.0	2.0	2.5	2.0
Tallinn	2.0	2.0	2.0	2.0	2.0
*Average (18 cities)*	*1.9*	*1.3*	*1.5*	*2.0*	*1.4*
*Average City* (*C*_*AV*_)	*1.9*	*1.3*	*1.4*	*2.6*	*1.5*

a The minimum is zero based on the samples with partial points for monitoring without an action plan.

b Top points received by DH/C based on CHP with >75% penetration and renewable energy, see Table A1.

c Scored based on sub-indicators for nearly net-zero energy buildings or districts implementation, see Table A2.

d Based on urban rail density, daily usership, and decentralized options with bicycle sharing (see Table A3).

e Penetration of LED armatures using solar energy and/or best practices obtain an extra point.

### 2.3 Data compilation on renewable energy potential and utilization

Table 5 represents the data acquisition process for data inputs into the main indicators of the dimension on “Renewable Energy Potential and Utilization” (*D*_*3*_). The data inputs that are compiled are necessary to evaluate the prevalence of renewable energy potential in cities while requiring cities to utilize higher shares of renewable energy for replacing the combustion of high exergy fossil fuels, especially in the electricity and transport sectors. The data acquisition process requires data inputs on the annual mean solar energy potential based on solar insolation on an optimally inclined plane, the average wind speed at 50 m height, and the mean heat-flow density for geothermal energy. The data acquisition process also extends to the renewable energy share in electricity generation to distinguish cities that have progressed towards or reached 100% renewable electricity grids with or without progress for decarbonizing the transport sector. The latter aspect requires data compilations for the share of green energy in transport, including biofuels and/or electricity.

**Table 5:** Data inputs to the renewable energy potential and utilization dimension (*D*_*3*_).

Indicators per City (*C*_*j*_)	*i*_3.1_	*i*_3.2_	*i*_3.3_	*i*_3.4_	*i*_3.5_
	Solar energy potential (Wh/m^2^/day)^a^	Wind energy potential (m/s)^a^	Geothermal energy potential (mW/m^2^)^b^	Renewable energy in electricity production (%)^c^	Green energy in transport (%)^d^
Data Sources	[100]	[101]	[102]	[103]	[104]
Aalborg	3550	7.0	65	56.00	6.58
Birmingham	3410	4.9	40	25.60	2.80
Bologna	4650	3.7	40	37.27	2.90
Cape Town	6110	6.4	65	4.18	0.00
Christchurch	3888	5.2	45	83.99	0.19
Constanţa	4710	6.0	40	46.20	3.20
Dublin	3460	5.9	65	25.00	2.40
Funchal	5770	5.5	70	10.00	0.00
Gdynia	3610	6.5	40	15.45	4.50
Glasgow	3020	5.3	65	25.60	2.80
Hamburg	3430	5.4	65	30.05	5.10
Johannesburg	6240	3.8	55	4.18	0.00
Murcia	5830	4.2	65	40.08	4.68
Reykjavík	2640	8.0	310	100.00	2.50
Riga	3440	6.2	40	55.00	3.44
Sfax	6180	5.1	70	3.00	0.00
Sydney	5000	7.0	71	16.86	0.70
Tallinn	3290	5.9	40	11.00	0.80
*Average (18 cities)*	*4346*	*5.7*	*70*	*32.75*	*2.37*
*Average City* (*C*_*AV*_)	*4535*	*4.7*	*67*	*37.15*	*3.53*

a Based on coordinate entries in the PVGIS [100] or IRENA [101] databases, respectively.

b Based on geothermal heat-flow density categories in [102] and/or local sources.

c Based on the share of renewable energy in electricity production based on [103] and/or local sources.

d Based on biofuel and/or electricity in transport given at least a 45% renewable share [104] or local sources.

### 2.4 Data compilation on water usage and environmental quality

Data acquisition for the dimension on “Water Usage and Environmental Quality” (*D*_*4*_) requires data inputs that are related to the use and quality of water resources and cleaner air as well as any ecological surplus or deficit, which can have an impact on maintaining or harming environmental integrity. Table 6 represents the data acquisition process for *D*_*4*_ that includes water usage per capita based on the water footprint of domestic blue water consumption and the level of water quality that is given out of a score of 100. The main data source for the annual mean PM_10_ concentration was sufficient for all cities in Table 6 except Cape Town and Funchal that required additional data sources [105, 106]. Similarly, data acquisition for ecological footprint and biocapacity per capita were obtained from the main data sources and compared with additional studies, e.g. ecological footprints of other Australian cities or housing and food shares in urban ecological footprints [107].

**Table 6:** Data inputs to the water usage and environmental quality dimension (*D*_*4*_).

Indicators per City (*C*_*j*_)	*i*_4.1_	*i*_4.2_	*i*_4.3_	*i*_4.4_	*i*_4.5_
	Domestic water consumption per capita (m^3^)	Water quality index (/100)^a^	Annual mean PM_10_ concentration (μg/m^3^)^b^	Ecological footprint per capita (gha)	Biocapacity per capita (gha)
Data Sources	[108, 109]	[110, 111]	[95, 105, 106]	[112–114]	[112]
Aalborg	7.7	81.5	24.0	6.11	4.57
Birmingham	3.5	90.5	18.5	4.72	1.27
Bologna	14.0	95.7	24.9	4.50	1.05
Cape Town	8.6	66.3	32.4	3.37	1.11
Christchurch	26.1	99.4	20.9	5.11	10.05
Constanţa	7.7	70.7	36.9	2.63	2.69
Dublin	6.7	79.3	15.7	4.80	3.69
Funchal	10.5	91.7	20.1	3.87	1.53
Gdynia	5.5	80.8	16.2	4.27	1.99
Glasgow	3.5	90.5	22.9	4.72	1.27
Hamburg	7.1	85.6	21.2	5.46	2.25
Johannesburg	8.6	66.3	85.3	3.37	1.11
Murcia	11.7	81.8	26.0	4.03	1.58
Reykjavík	17.7	57.0	15.1	6.40	22.13
Riga	6.8	97.6	34.0	6.53	9.50
Sfax	3.8	63.8	87.0	3.10	0.79
Sydney	18.2	85.2	16.6	7.00	15.67
Tallinn	6.6	76.4	14.0	7.01	10.24
*Average (18 cities)*	*9.7*	*81.1*	*29.5*	*4.83*	*5.14*
*Average City* (*C*_*AV*_)	*9.8*	*84.0*	*30.7*	*4.30*	*2.70*

a Based on UN water quality index for dissolved oxygen, pH, conductivity, nitrogen and phosphorus.

b The related concentration should be below an annual mean of 20 μg/m^3^ based on WHO guidelines [115].

### 2.5 Data compilation on carbon dioxide emissions and industrial profile

The data acquisition process for the dimension on “CO_2_ Emissions and Industrial Profile” (*D*_*5*_) as represented in Table 7 involves data inputs to assess the impacts of the urban system on carbon dioxide (CO_2_) emissions. The main data sources are sufficient to attain data inputs on the CO_2_ emissions of residential, tertiary, and municipal buildings as well as those of private vehicles, public transport, and the municipal vehicle fleet. The CO_2_ intensity is calculated to determine the level of decarbonization in urban sectors and the level of decoupling between energy usage and CO_2_ emissions. Data inputs extend to components of the urban system that are otherwise not required in regular emissions reporting. The data compilation for the presence of any energy-intense industries in urban and related port areas, including iron and steel, basic chemicals and chemical products are represented in Table A4. The implementation of measures for on-site energy generation from renewable energy sources in airports towards carbon neutrality is evaluated from the annual reports of airports that service each city also considering the Airport Carbon Accreditation (ACA) levels.

**Table 7:** Data inputs to the CO_2_ emissions and industrial profile dimension (*D*_*5*_).

Indicators per City (*C*_*j*_)	*i*_5.1_	*i*_5.2_	*i*_5.3_	*i*_5.4_	*i*_5.5_
	CO_2_ emissions of buildings (t CO_2_)	CO_2_ emissions of transport (t CO_2_)	Average CO_2_ intensity (t CO_2_/MWh)	Number of CO_2_ intense industries^b^	Airport ACA level and measures^c^
Data Sources	[12–52]^a^	[12–52]^a^	[12–52]^a^	Table A4	[116]
Aalborg	1,409,080	273,883	0.25	2	3
Birmingham	4,063,000	1,133,000	0.30	4	0
Bologna	1,441,075	332,733	0.29	2	2
Cape Town	10,751,843	6,974,396	0.47	6	1
Christchurch	893,000	1,735,000	0.27	2	1
Constanţa	242,272	94,696	0.21	4	2
Dublin	2,081,621	717,710	0.28	5	2
Funchal	249,246	144,483	0.36	1	2
Gdynia	737,540	103,427	0.42	2	0
Glasgow	2,138,300	845,200	0.27	4	0
Hamburg	7,571,000	3,459,000	0.31	7	4
Johannesburg	6,859,405	7,692,684	0.46	5	2
Murcia	683,386	642,168	0.31	3	0
Reykjavík	28,317	232,079	0.06	3	1
Riga	1,345,539	742,000	0.20	5	1
Sfax	223,070	309,371	0.31	2	0
Sydney	4,256,850	336,700	0.49	7	3
Tallinn	1,823,000	888,000	0.42	3	0
*Average (18 cities)*	*2,599,864*	*1,480,918*	*0.32*	*3.7*	*1.3*
*Average City* (*C*_*AV*_)	*2,275,621*	*1,093,392*	*0.29*	*3.5*	*1.4*

a Calculated from SEAP, SECAP or equivalent plans based on references in Table 1 [12–52].

b Includes sectors that require high-temperature processes (e.g. kiln heating up to 2000 °C) [117], see Table A4.

c Scores greater than 3 require renewable energy best practices on the land side, air side and/or ground side.

### 2.6 Data compilation on urban planning and social welfare

The process of acquiring data to evaluate the provision of liveable areas with high levels of social welfare is based on the data sources of the indicators for the dimension on “Urban Planning and Social Welfare” (*D*_*6*_). The data inputs in Table A5 are used to evaluate aspects of the waste hierarchy based on waste generation per capita and the share of waste that is recycled, reused or composted. Table A6 provides data inputs for any share of discharge without treatment and compliance with biochemical (BOD) and chemical oxygen demand (COD) as well as total suspended solids (TSS) in the wastewater treatment infrastructure primarily based on the thresholds of UWWTD [118]. In addition to urban services for waste and wastewater management, Table A7 provides data inputs based on the share of the population that lives in core urban areas, the sprawl index, the share of green urban areas, and protected green areas in the vicinity. The multiple data inputs compare urban compactness with the presence of green areas in support of ecological services [119] and climate adaptation [120]. Economic and educational opportunities are evaluated based on other data inputs as represented in Table 8, namely gross domestic product (GDP) per capita if distributed equally in society, inequality-adjusted well-being depending on survey results for daily experience satisfaction, including employment, and the tertiary education rate.

**Table 8:** Data inputs to the urban planning and social welfare dimension (*D*_*6*_).

Indicators per City (*C*_*j*_)	*i*_6.1_	*i*_6.2_	*i*_6.3_	*i*_6.4_	*i*_6.5_
	Waste and wastewater management^a^	Compact urban form and green spaces^b^	GDP per capita (PPP$ national)	Inequality-adjusted well-being (/10)	Tertiary education rate (%)
Data Sources	[96, 97, 121–125]	[126–129]	[130]	[131]	[132–134]
Aalborg	5.5	2.7	49,696	7.9	47.7
Birmingham	5.0	2.3	36,465	7.4	48.1
Bologna	5.4	2.0	38,161	7.1	26.2
Cape Town	3.8	2.7	13,225	7.3	7.0
Christchurch	5.3	1.7	39,059	7.6	46.0
Constanţa	4.6	1.7	23,626	6.6	25.6
Dublin	4.1	1.7	68,883	7.5	52.9
Funchal	4.1	1.7	30,624	7.1	34.6
Gdynia	5.4	2.0	27,811	7.1	44.6
Glasgow	5.2	2.0	42,609	7.4	59.9
Hamburg	5.3	2.0	48,730	7.4	33.2
Johannesburg	4.1	2.0	13,225	7.3	7.0
Murcia	5.2	2.0	36,310	7.0	40.1
Reykjavík	5.3	2.0	51,399	8.2	41.0
Riga	4.5	2.0	26,031	6.5	42.8
Sfax	3.4	1.3	11,599	6.8	11.9
Sydney	5.2	2.3	46,790	7.5	45.0
Tallinn	5.7	1.7	29,365	6.8	45.4
*Average (18 cities)*	*4.8*	*2.0*	*35,200*	*7.3*	*36.6*
*Average City* (*C*_*AV*_)	*4.6*	*1.9*	*31,152*	*6.9*	*34.3*

a Based on municipal waste management and wastewater treatment sub-indicators (Tables A5-A6).

b Based on compact urban form including sprawl index and green spaces sub-indicators (Table A7).

### 2.7 Data compilation on research, innovation and sustainability policy

The process of acquiring data for dimension *D*_*7*_ on “Research and Development (R&D), Innovation and Sustainability Policy” involves at least seven data sources for cross-cutting data inputs on the alignment of R&D and innovation assets in support of sustainable energy, transport, water and environment systems (Table 9). The sub-indicators are based on R&D and innovation policy orientation (Table A8) and national patents in clean technologies based on Y02 and Y04 coded patents (Table A9). Both sub-indicators also require additional data acquisition, including patents in building technologies, energy generation, transport, smart grid and carbon capture and storage to determine technological competences [135]. Other data inputs are based on the presence of higher education and research institutions in the city (Table A10), the knowledge production capacity based on the *h*-index, and the emissions mitigation target as a major target for sustainability policy. Targets beyond the year 2020 towards carbon neutrality are annualized to 2020 for a common basis.

**Table 9:** Data inputs to the R&D, innovation and sustainability policy dimension (*D*_*7*_).

Indicators per City (*C*_*j*_)	*i*_7.1_	*i*_7.2_	*i*_7.3_	*i*_7.4_	*i*_7.5_
	R&D and innovation policy orientation^a^	National patents in clean technologies^b^	Universities/institutes in the local ecosystem^c^	National *h*-index^d^	Reduction target for CO_2_ Emissions
Data Sources	[136, 137]	[138]	[139]	[140]	[54, 55]
Aalborg	3.0	2.0	2	619	40
Birmingham	2.0	2.5	5	1213	32
Bologna	2.0	2.0	2	839	20
Cape Town	2.0	2.0	6	361	13
Christchurch	1.5	2.0	4	428	20
Constanţa	1.5	1.0	2	201	20
Dublin	2.5	1.5	12	414	20
Funchal	2.0	2.0	2	379	21
Gdynia	2.0	1.5	2	445	20
Glasgow	2.0	2.5	8	1213	30
Hamburg	3.0	3.0	7	1059	25
Johannesburg	2.0	2.0	6	361	22
Murcia	1.5	2.0	4	723	20
Reykjavík	2.0	1.0	4	251	33
Riga	1.5	2.0	4	129	55
Sfax	1.0	0.0	2	144	20
Sydney	3.0	2.0	14	795	35
Tallinn	2.0	1.0	4	215	20
*Average (18 cities)*	*2.0*	*1.8*	*5*	*544*	*26*
*Average City* (*C*_*AV*_)	*2.1*	*1.7*	*8*	*492*	*24*

a Based on the approach for thematic priorities and R&D expenditure as a share of GDP (Table A8).

b Patents are limited to clean energy technology coded patents, e.g. Y02B for buildings etc. (Table A9).

c Sum of universities located in the city. Those in the Scimago list receive double points (Table A10).

d Sustainable development is a multidisciplinary field with inputs from multiple fields (fields not restricted).

### 2.8 Data analyses based on the compiled data inputs

As indicated in the specifications table, formatted data that takes place in Table 1, Table 2, Table 3, Table 4, Table 5, Table 6, Table 7, Table 8, Table 9, Table 10 and Tables A1-A10 in Appendix A require processing and analysis. The analysis of the compiled data inputs for 120 cities are undertaken as described in the research article [1] based on the determination of the presence of any outlier values according to higher order moments, normalization based on the Min-Max method, uncertainty analyses based on 10,000 Monte Carlo simulations and sensitivity analyses based on various schemes using linear aggregation and/or aggregation based on the geometric mean at the dimension and index levels. These experimental factors led to the use of winsorized values for minimum or maximum values in at least one indicator (*i*_*x.y*_) in *D*_*1*_ (*i*_*1.1*_*-i*_*1.3*_), *D*_*3*_ (*i*_*3.3*_*, i*_*3.5*_), *D*_*4*_ (*i*_*4.3*_*, i*_*4.5*_), *D*_*5*_ (*i*_*5.1*_*, i*_*5.2*_), and *D*_*7*_ (*i*_*7.3*_*, i*_*7.5*_) as shared in this data article. In particular, these indicators include those on energy usage per capita (Washington D.C. from the benchmarking in [4]), PM_10_ (e.g. Cape Town and Johannesburg from the newly benchmarked cities and Bangalore from [6]), and biocapacity per capita (Helsinki and Espoo from [4], [6]). The winsorized values in the scope of these indicators have been included in the values for *C*_*AV*_.

Data acquisition through data compilation, data processing, and the process of performing data analysis accumulates into the composite indicator. The normalized values with linear aggregation at the dimension level are provided in Appendix B. The relevant values are provided for the top 30 cities as the pioneering cities in Table B1, the top 31–60 cities as the transitioning cities in Table B2, the lower 61–90 cities as the solution-seeking cities in Table B3 and the lower 91–120 cities as the challenged cities in Table B4. Moreover, the compiled and analyzed data sets for 120 cities are processed to create a SDEWES Index Atlas. In this atlas, a 3-D maps feature is used to establish layers that are juxtaposed on Google Maps by dimensions and the index value per city for each group of 30 cities as represented in Fig. 1. The atlas supports another means in which the index results are used for city comparisons, including pairs as given in the research article [1].

### 2.9 Data compilation on residual energy in the urban vicinity

As indicated in the specifications table, data acquisition was further required for an original scenario application to cities. In the context of this cross-sectoral scenario as put forth in the research article [1], Table 10 represents the process of data acquisition that took place separately for 60 cities based on the values of the theoretically available residual heat from the industry and thermal power generation in about a 15 km radius based on spatial data in local maps [7], [8]. Data related to wastewater and urban biowaste also take place in Table 10. According to the method of the companion research article [1], scenario multipliers are applied to these theoretical amounts to obtain scenario values after which the index is re-calculated for comparative analysis.

**Table 10:** City level data compilation for theoretical potentials of residual energy.

Cities^a^, ^b^	Theoretical Potentials of Residual Heat							Biowaste (MWh/a) e
	T. Power Gen.		Industrial Sector Groups c			Total Value (MWh/a) d	Wastewater (MWh/a) e	
	MA	WtE	Group 1	Group 2	Group 3			
Aalborg	✓	✓	✓			2,061,111	N/A	88,889
Amsterdam	✓	✓	✓		✓	26,069,444	N/A	566,667
Antwerp	✓	✓	✓	✓		20,600,000	N/A	358,333
Århus	✓	✓				3,063,889	N/A	166,667
Barcelona	✓	✓	✓			5,258,333	N/A	2,038,889
Bari	✓					1,619,444	108,889	325,000
Berlin	✓	✓	✓			11,430,556	N/A	1,250,000
Bilbao	✓	✓	✓	✓	✓	7,047,222	N/A	452,778
Birmingham		✓				1,197,222	993,056	97,222
Bologna		✓				352,778	136,111	247,222
Braşov	✓					522,222	49,444	150,000
Bregenz						0	N/A	125,000
Bucharest	✓					4,166,667	386,389	577,778
Budapest	✓	✓				2,441,667	N/A	338,888
Byggoszcz	✓					1,066,667	N/A	169,444
Cluj-Napoca						0	52,778	152,778
Cologne	✓	✓	✓	✓	✓	38,888,889	N/A	363,889
Constanţa	✓					619,444	47,778	150,000
Copenhagen	✓	✓				3,725,000	N/A	194,444
Dubrovnik						0	4444	N/A
Espoo	✓					4,425,000	N/A	455,556
Florence	✓		✓			263,889	270,556	277,778
Frankfurt	✓	✓				4,652,778	N/A	244,444
Gdynia	✓					1,000,000	N/A	186,111
Genoa	✓					1,519,444	190,833	280,556
Glasgow						0	563,611	191,667
Gothenburg	✓	✓		✓		3,619,444	N/A	322,222
Grand Lyon		✓	✓	✓		3,525,000	N/A	452,778
Grenoble	✓	✓	✓			1,127,778	N/A	263,889
Hamburg	✓	✓	✓	✓		10,877,778	N/A	650,000
Helsinki	✓					4,425,000	N/A	455,556
Karlovac	✓		✓		✓	36,111	10,000	N/A
Klagenfurt					✓	166,667	N/A	113,889
Leuven					✓	47,222	N/A	172,222
London	✓	✓			✓	6,386,111	3,997,778	319,444
Madrid	✓	✓				825,000	N/A	2,347,222
Málaga			✓			738,889	N/A	244,444
Milan	✓	✓				2,522,222	2,357,778	994,444
Murcia						0	N/A	361,111
Naples	✓	✓				2,591,667	993,056	1,022,222
Nice		✓	✓			1,202,778	N/A	277,778
Osijek	✓		✓		✓	266,111	17,222	N/A
Ostrava	✓		✓	✓		6,147,222	200,833	269,444
Paris	✓	✓				8,097,222	N/A	655,556
Pécs	✓					1,150,000	N/A	47,222
Pisa			✓			172,222	81,667	105,556
Pula			✓			88,889	11,667	N/A
Rijeka				✓		900,000	36,389	N/A
Rome	✓	✓		✓		1,125,000	675,000	1,166,667
Sevilla			✓			347,222	N/A	594,444
Stockholm	✓	✓				3,338,889	N/A	497,222
Timișoara	✓					572,222	50,278	133,333
Turin	✓					3,700,000	490,833	677,778
Valencia	✓					2,369,444	N/A	852,778
Venice	✓	✓	✓	✓		8,658,333	31,389	219,444
Vienna	✓	✓		✓		8,555,556	N/A	750,000
Warsaw	✓	✓				8,363,889	N/A	441,667
Zadar					✓	2778	9444	N/A
Zagreb	✓		✓	✓	✓	1,921,167	170,000	N/A
Zaragoza		✓			✓	1,066,667	N/A	280,556

a Data for Croatian cities take place in the STRATEGO local maps [8] from which the data is compiled.

b The values are summed and converted from units of PJ or TJ to units of MWh per annum as needed.

c Grouped for convenience as Sector 1 (iron/steel; non-ferrous metals; non-metallic minerals), Sector 2 (chemical/petrochemical; fuel supply/refineries) and Sector 3 (paper/pulp/printing; food/tobacco).

d The total of theoretically available residual heat from thermal power generation and industrial sector groups.

e Available heat from wastewater and the amount of biowaste as one of the options under “technical and economically available biomass” are included in STRATEGO local maps [8] and Peta 4.2 maps [7], respectively.

Hence, the above process up to Table 10 supports the counterpart research article [1] in the capacity of data acquisition as well as details of the method for processing and analyzing the data inputs.

## Footnotes

1 References [12–53] and subsequent references are provided in a third appendix to comply with journal formatting.

2 The questionnaire is available from the author.
