# Supplementary material for: Data on cities that are benchmarked with the sustainable development of energy, water and environment systems index and related cross-sectoral scenario
Source: Data Brief. 2019 Mar 20;24:103856. doi: 10.1016/j.dib.2019.103856 (PMC6538927; doi:10.1016/j.dib.2019.103856)
Supplement: Multimedia component 1 [file mmc1.pdf]

Conflict of Interest and Authorship Confirmation Form

The following has been marked as appropriate.

☒ The author has undertaken (a) conception and design, analysis and interpretation of the data; (b) drafting the article or revising it critically for important intellectual content; and (c) approval of the final version.

☒ The article that I have submitted to the journal for review is original, has been written by the stated author and has not been published elsewhere.

☒ The images that I have submitted to the journal for review are original, were developed by the stated author, and have not been published elsewhere.

☒ This manuscript has not been submitted to, nor is under review at, another journal or other publishing venue.

☒ The author has no affiliation with any organization with a direct or indirect financial interest in the subject matter discussed in the manuscript

☐ The below authors have affiliations with organizations with direct or indirect financial interest in the subject matter discussed in the manuscript:

N/A
