# Supplementary material for: Data on cities that are benchmarked with the sustainable development of energy, water and environment systems index and related cross-sectoral scenario
Source: Data Brief. 2019 Mar 20;24:103856. doi: 10.1016/j.dib.2019.103856 (PMC6538927; doi:10.1016/j.dib.2019.103856)
Supplement: Multimedia component 2 [file mmc2.pdf]

Appendix A. Supplementary material.

This appendix is based on Tables A1 to A10 that involves references [142] to [183] of the data article on “Data on cities that are benchmarked with the Sustainable Development of Energy, Water and Environment Systems Index and related cross-sectoral scenario” in association with [1].

Table A1. Energy System Characteristics Based on Original Compilations

**City (C_j)**	HOB/ electric HVAC or DH only	CHP based DH/C	Geo-thermal/ solar, GHSP, seawater HP	Integration of other sources (waste heat)	Renewable energy to hydrogen (P2G)	
Aalborg		✓[a]				DH based on waste heat from waste-to-energy plant (Reno Nord), Aalborg Portland, and Nordjylland power stations [142]
Birmingham		✓	✓[b]			Birmingham District Energy Scheme involves 56 GWh of heat, 51 GWh of electricity, and 8 GWh of chilled water p.a. [143]
Bologna		✓		✓[c]		Includes Casalegno and 4 other CHP plants for DH that produced 314 GWh of electricity and 137 GWh of thermal energy [18]
Cape Town	✓					Ankerlig OCGT Power Station SA is based on diesel to supply electricity to the grid during peak hours with 1338 MW_{el} [144]
Christchurch	✓	P	✓[d]	✓		DH system is proposed with RE while 10 MW local energy production includes 1.8 MW from WWT and landfill biogas [23]
Constanța		✓[e]				Electrocentrale Constanta provides 100 MW_{el} and 140 MW_{th} as CHP while other thermal power plants of 31 MW_t exist [145]
Dublin	✓	P		✓[f]		75% of Dublin is suitable for DH in which the Dublin DH scheme is planned to replace the use of natural gas in HOB [146]
Funchal	✓					Thermoelectric plants based on fuel oil/natural gas have a dominant share in the energy system of Madeira Island [147]
Gdynia	✓	✓		✓[g]		The heat only DH of Wejherowo that services Gdynia is partially transformed to CHP with 6 MW_{th} and 6.7 MW_{el} outputs [148]
Glasgow	✓	P				RE CHP and DH was 1% of energy use, e.g. CHP of university and Athletes Village, with gas or electric heating dominant [33]
Hamburg		✓	✓	✓	✓	Reiherstieg district is supplied by 100% RE and Hamburg Energie provides green electricity and 5% urban biogas share [149]
Johannesburg	✓					Kelvin A and B Thermal Power Station is based on bituminous coal with 600 MW_{el} with CHP uses in data centers only [150]
Murcia		✓			✓[h]	CHP represents 308 MW_{el} of installed power, including biogas based on the waste plant Cañada Hermosa de Murcia [151]
Reykjavík		✓	✓			Geothermal energy based DH provides a total of 10,922 TJ to urban areas of which 10,041 TJ is for space heating [44]
Riga		✓		✓[i]		Riga TEC-1 and 2 produced 2,206 GWh_{el} of power and 2,417 GWh_{th} of thermal energy with 88% and 89% efficiency [45]
Sfax	✓					Tertiary buildings use largely electricity, residential buildings use electricity, gas, and LPG, municipality buildings use gas [46]
Sydney	✓	✓[j]	P	P	P	Plans to displace 2,365 GWh of coal-fired electricity with gas and/or thermal networks and 537 TJ from energy savings [48]
Tallinn		✓		P[k]		Väo Power Plant has capacities of 25 MW_{el} and 67 MW_{th} uses biomass and peat with a new second station in operation [152]

Table A1 (Continued). Details of Power-to-Gas Pilot Demonstration Projects [153] [l]

**City (C_j)**	Installed power (kW)	Power load	H_2 production (Nm^3/h)	Electrolysis	Application	Current Status
Hamburg	1000	Flexible	290	PEM	Gas grid	Operational
Hamburg	630	Base	120	Alkaline	Mobility	Operational
Hamburg	185	Flexible	30	PEM	Mobility	Operational

Notes to Table A1:

[a] The current energy mix of Aalborg also includes coal while nearby Brønderslev transitioned to concentrated solar power and biomass with an organic Rankine cycle [154]
[b] An urban development area with a complex of shops, offices restaurants and apartments uses a canal based cooling system [16]
[c] Energy recovery from water treatment sludge as biogas and other waste-to-energy plants are operational with a biomethane plant from organic waste planned for 2018 [155]
[d] The airport uses 12°C groundwater for cooling and heating that replaced the old diesel and LPG boilers. The city has geothermal springs less than 90°C [156]
[e] Currenty based on natural gas and fuel oil while cogeneration with biogas from the sludge of wastewater treatment plants is among planned measures [25]
[f] Ringsend WWTW captures methane gas to generate 40% of its electrical loads and a majority of the steam heating needs [26]
[g] Dębogórze Wastewater Treatment Plant (PEWIK Gdynia) operates a 600 kW CHP system for the use of the waterworks [157]
[h] A PEMFC unit treating 10 m^3/h biogas from the WWTP of Murcia produces 3 kW_e based on methane dry reforming process [158]
[i] A project captures waste heat from wastewater to supply heat to multi-apartment residential buildings in the city [45]
[j] A trigeneration scheme for 11 high rise buildings (2,100 apartments) in the Central Park district energy scheme is expanding with an additional 1.1 MW_e [159]
[k] AS Tallinna Vesi Paljassaare WWT plant already uses anaerobic sludge digestion and the use of the residual heat in the wastewater is planned [52]
[l] The examples from Hamburg support the NEW (North German Energy Transition) 4.0 project concept with 300 MW flexibility, storage, and 1600 MW virtual power plants that involve at least 4.3 MW electrolysers [160]

Table A2. Sub-Indicators for nZEB/Districts/Port Implementations in Cities

C_j	National nZEB Plan [161]	Scope of nZEB Definition [161-163]			nZEB implementation and/energy plus / carbon neutral buildings/district targets
		New Buildings	Existing Buildings	Minimum RE Share	
Aalborg	✓ [a]	✓	✓	✓(56%)	□ Port of Aalborg is carbon neutral (40% less energy and 2 MW RE) [164]
Birmingham	✓	✓	✓	✓	□ Zero Carbon House based on the Code for Sustainable Homes [165]
Bologna	✓	✓	✓	✓	□ Nearly zero-energy buildings of the Ozzano development [166]
Cape Town	[b]				□ Greenfield Industrial Park in Airport Industria will be a net-zero pilot [167]
Christchurch					□ Civic Offices building as net zero carbon public building [168]
Constanța	✓	✓	✓		-
Dublin	✓	✓	✓	✓	□ Carbon Neutral Housing and Dublin City Council housing [169]
Funchal	✓	✓	✓		-
Gdynia	✓	✓	✓	✓	-
Glasgow	✓	✓	✓	✓	□ Clyde Waterfront water source heat pump medium temperature DH [170]
Hamburg	✓	✓	✓	✓(60%)	□ Energy Bunker at Reiherstieg district for self-sufficiency [171]
Johannesburg	[b]				-
Murcia	✓	✓		✓	-
Reykjavík					-
Riga	✓	✓	✓	✓	-
Sfax					-
Sydney	[b]				□ Net zero challenge adoption and net-zero high-rise apartment buildings [172, 173]
Tallinn	✓	✓	✓	✓	□ Near-zero energy test building of Tallinn University of Technology [174]

[a] Denmark indicates the primary energy performance to be 20 kWh/m^2 for heating, cooling, domestic hot water, ventilation and lighting in residential buildings in 2020 [175]
[b] The Green Building Councils of South Africa (GBCSA) and Australia (GBCA) as well as WGBC pledged to introduce net-zero building certification at COP21 [176]

Table A3. Sub-Indicators for the Density of the Public Transport System

C_j [a]	Bus/ trolley bus lines	Trams	Subway/Metro	Total length urban rail (km)	Total urban area (km^2) [177]	Urban rail density (km/km^2)	Daily Ridership per km [178]	Municipal Bicycle Sharing		
		Length (km)	*Length (km)*					Program	Stations	Bicycles
Aalborg	✓			122	121.6	0.00		✓		
Birmingham	✓	21		599	598.9	0.03		✓		
Bologna	✓		P (70) [b]	155	140.9	0.00		✓ [f]	90	
Cape Town	✓			816	400.3	0.00		✓		
Christchurch	✓	3.9		202	607.7	0.00		✓		
Constanța	✓			125	124.9	0.00		✓		
Dublin	✓	36.5	P (16.5)	318	318.0	0.12	2330	✓	102	950
Funchal	✓ [c]			76.2	76.2	0.00				
Gdynia	✓			135	135.0	0.00		✓		
Glasgow	✓		10	368	174.7	0.03	3420	✓	31	400
Hamburg	✓ [d]		116.8	777	755.0	0.13	5210	✓	102	1250
Johannesburg	✓			-	334.8	0.00				
Murcia	✓	17.5		98	882.0	0.18		✓		
Reykjavík	✓			80	273.0	0.00		✓		
Riga	✓	99.53		324	324.0	0.56	436	✓	20	
Sfax	✓			199	140.0	0.00				
Sydney	✓ [e]	12.8	P (65)	2037	12368	0.01		✓		
Tallinn	✓	39		181	159.2	0.22				

[a] Cities that have tramways or subways are further evaluated based on urban rail density. Scores higher than 3 are given to densities above 0.10 km/km^2 and/or multiple modes.
[b] Represents the length for the project in the urban context of Bologna within the greater Bologna Metropolitan Rail Service (SFM) [179]
[c] The city has an electrical bus line that runs in the city center (Linha Eco) [180]
[d] In total 68 buses with alternative propulsion (hybrid) with 11 hydrogen fuel cell buses are deployed in the public transport system mainly in the Innovationslinie 109 [181]
[e] In addition, a CBD and South East line is being constructed to reduce the bus congestion [182]
[f] An example of bicycle sharing programs may be given from "C'entro in Bici" that supports the pedestrian zones in the historical city center [183]

Table A4. Evaluation of energy intensive industries in the cities

Presence of energy intensive industries in the cities [a, b]	Aalborg	Birmingham	Bologna	Cape Town	Christchurch	Constanța	Dublin	Funchal	Gdynia	Glasgow	Hamburg	Johannesburg	Murcia	Reykjavik	Riga	Sfax	Sydney	Tallinn
Basic chemicals and chemical products	1	1	1	1	1	1	2		1	1	2	2	1		1	2	2	2
Basic precious and non-ferrous metals		1		1										2	2		1	
Cement, lime and plaster industry	1	1										1	1	1	1			
Ceramic products industry		1		1			2	1										
Iron and steel industry				1	1	1			1	1	2	1	1				2	
Pulp, paper and paperboard industry			1							1	1	1						
Refined petroleum products industry				2		2				1	2				1		1	1

[a] The presence of at least one large enterprise/factory in the sector receives a binary value of 1
[b] The presence of clustered industries in the sector receive a binary value of 2

Table A5. Sub-indicators for municipal waste management

Municipal Waste Management	Waste per Capita (kg) [121-123]	Reuse, Recycling or Composting (%) [121-123]	Total Scoring Waste Management [a]
Aalborg	380	36	2.5
Birmingham	436	27	2.0
Bologna	562	45	2.4
Cape Town	573	31	1.8
Christchurch	524	40	2.3
Constanța	247	7	1.7
Dublin	264	20	2.1
Funchal	452	30	2.1
Gdynia	286	29	2.4
Glasgow	482	37	2.3
Hamburg	482	38	2.3
Johannesburg	440	7	1.2
Murcia	434	33	2.2
Reykjavík	345	31	2.4
Riga	486	18	1.6
Sfax	260	5	1.6
Sydney	385	30	2.2
Tallinn	481	49	2.7
Average (18 cities)	418	29	2.1
Average (sample of 120 cities)	433	27	2.0

[a] Sum of terms based on the top score minus the ratios of city values over the average values of 433 kg per capita and 26.8% for the sample

Table A6. Sub-indicators for municipal wastewater management

Municipal Wastewater Management	Discharge without treatment	Percentage of Compliance (x 100) [a] [98, 99, 124, 125]			Scoring Coverage [b]	Scoring Compliance [c]	Total Scoring
		BOD	COD	TSS			
Aalborg	0.00	1.00	1.00	1.00	2.00	1.00	3.00
Birmingham	0.00	1.00	1.00	1.00	2.00	1.00	3.00
Bologna	0.00	1.00	1.00	1.00	2.00	1.00	3.00
Cape Town	0.31	0.92	0.92	0.92	1.08	0.92	2.00
Christchurch	0.00	1.00	1.00	1.00	2.00	1.00	3.00
Constanța	0.01	1.00	1.00	1.00	1.97	1.00	2.97
Dublin	0.00	0.00	0.00	0.00	2.00	0.00	2.00
Funchal	0.00	0.00	0.00	0.00	2.00	0.00	2.00
Gdynia	0.00	1.00	1.00	1.00	2.00	1.00	3.00
Glasgow	0.00	1.00	1.00	1.00	2.00	1.00	3.00
Hamburg	0.00	1.00	1.00	1.00	2.00	1.00	3.00
Johannesburg	0.00	0.91	0.91	0.91	2.00	0.91	2.91
Murcia	0.00	1.00	1.00	1.00	2.00	1.00	3.00
Reykjavík	0.00	1.00	1.00	1.00	2.00	1.00	3.00
Riga	0.00	1.00	1.00	1.00	2.00	1.00	3.00
Sfax	0.06	0.00	0.00	0.00	1.81	0.00	1.81
Sydney	0.00	1.00	1.00	1.00	2.00	1.00	3.00
Tallinn	0.00	1.00	1.00	1.00	2.00	1.00	3.00
Average (18 cities)	0.02	0.82	0.82	0.82	1.94	0.82	2.76
Average (sample of 120 cities)	0.06	0.80	0.80	0.80	1.83	0.80	2.64

[a] When there is more than one plant, percentage compliance is weighted by the total wastewater load of the urban area
[b] Coverage scoring includes a penalty multiplier for any discharge without treatment subtracted from the top score
[c] Compliance scoring is the average of the relevant criteria for BOD, COD and/or TSS divided by 100

Table A7. Sub-indicators for Compact Urban Form and Green Areas

Urban Form and Municipal Management	Aalborg	Birmingham	Bologna	Cape Town	Christchurch	Constanța	Dublin	Funchal	Gdynia	Glasgow	Hamburg	Johannesburg	Murcia	Reykjavík	Riga	Sfax	Sydney	Tallinn
**Compact urban form (1-3)** [a]	**2**	**3**	**2**	**2**	**1**	**1**	**1**	**1**	**2**	**2**	**2**	**1**	**2**	**2**	**1**	**1**	**2**	**1**
• *Polycentricity (> 1 core area)* [126]		✓ (2)								✓ (2)		✓	✓	✓			✓ (2)	
• *Population core areas* (%) [126]		88	49				75		63	63	57						87	75
• *Sprawl index* (%) [126]		-3.97	-0.1	1.7			5.9			-0.4	1.5	5.4						9.1
**Urban green space (1-3)** [b]	**3**	**2**	**2**	**3**	**2**	**1**	**2**	**2**	**2**	**2**	**2**	**2**	**2**	**2**	**2**	**1**	**2**	**2**
• *Urban park intensity*	✓	✓	✓	✓	✓		✓	✓	✓	✓	✓	✓	✓	✓	✓		✓	✓
• *Percentage green areas* (%) [127]	30.32	20.31	24.3	40.5	20.0	13.36	21.58	17.7	22.49	23.7	22.21	24.0	31.2		26.75	N/A	14.8	27.08
• *Impermeable surfaces* (%) [120]	26.39	43.38				65.66	50.41		57.62		48.48				43.73			47.13
• *Green area per capita* (m^2) [126]		138.31	183.8	289.5			19.52		671.47	103.43	914.18	230.7		270		14.5	46.47	1469.15
**Green corridor quality (1-3)**	**3**	**2**	**2**	**3**	**2**	**3**	**2**	**2**	**2**	**2**	**2**	**3**	**2**	**2**	**3**	**2**	**3**	**2**
• *Natural reserves* [128]	5	4	6	1	2	5	1	1	5	2	5	4	7	2	6	1	1	6
• *RAMSAR* [129]	0	3	5	1	0	3	6+	0	2	3	2	1	6	2	2	4	1	2
• *National park* [128]	0	0	0	1	0	0	1	1	1	1	0	0	0	1	2	0	5	1
• *Total number evaluation*	5	7	11	3	2	8	8+	2	8	6	7	5	13	5	10	5	7	9
• *Total area > 2,700 km²*				✓		✓												
Average category score	**2.7**	**2.3**	**2.0**	**2.7**	**1.7**	**1.7**	**1.7**	**1.7**	**2.0**	**2.0**	**2.0**	**2.0**	**2.0**	**2.0**	**2.0**	**1.3**	**2.3**	**1.7**

[a] Scored so that polycentricity, high share of the population living in core urban areas, and sprawl index far less than 0 receive the top score. Additional reports are taken into account.
[b] The best practice score of 3 is given to cities with about a 40% or more share of green areas [127] and/or less than 30% share of impermeable surfaces [120] based on data availability.

Table A8. Sub-indicators for benchmarking R&D and innovation policy orientation (I)

R&D and innovation policy orientation [a]	AU	DE	DK	EE	ES	GB	IE	IS
R&D funding approach score	**3**	**3**	**3**	**2**	**1**	**2**	**3**	**1**
General(no thematic focus)	✓	✓	✓	✓	✓	✓	✓	✓
Thematic focus (calls)	✓	✓	✓				✓	
Energy environment /smart cities priority	✓	✓	✓	✓		✓	✓	
R&D expenditure score	**3**	**3**	**3**	**2**	**2**	**2**	**2**	**3**
GERD/GDP (percentage)	2.20	2.92	2.98	1.50	1.3	1.72	1.51	2.22
Average category score	**3**	**3**	**3**	**2**	**1.5**	**2**	**2.5**	**2**

[a] Policy scan involves R&D funding institutions, support mechanisms, and country reports from JRC [136] and OECD/UNESCO [137]

Table A8. Sub-indicators for benchmarking R&D and innovation policy orientation (II)

R&D and innovation policy orientation [a]	IT	LV	NZ	PL	PT	RO	TN	ZA
R&D funding approach score	**2**	**2**	**2**	**2**	**2**	**2**	**1**	**3**
General(no thematic focus)	✓	✓	✓	✓	✓	✓	✓	✓
Thematic focus (calls)								✓
Energy environment /smart cities priority	✓	✓	✓	✓	✓	✓		✓
R&D expenditure score	**2**	**1**	**1**	**2**	**2**	**1**	**1**	**1**
GERD/GDP (percentage)	1.29	0.63	1.15	1.0	1.5	0.39	0.63	0.72
Average category score	**2**	**1.5**	**1.5**	**2**	**2**	**1.5**	**1**	**2**

[a] Policy scan involves R&D funding institutions, support mechanisms, and country reports from JRC [136] and OECD/UNESCO [137]

Table A9. Sub-indicators for benchmarking national patents in clean technologies (I)

National patents in clean technologies	AU	DE	DK	EE	ES	GB	IE	IS
Total Y02 or Y04 patents [a]	18,817	146,946	7,508	138	21,699	27,627	623	87
Building technologies (Y02B)	7,683	21,158	1,249	37	3,139	6,340	125	12
Energy generation (Y02E)	10,000+	60,958	4,762	77	12,535	10,000	382	61
Transportation (Y02T)	8,023	60,961	1,140	16	5,179	10,000	85	9
Capture and storage (Y02C)	1,820	1,448	211	4	469	578	15	3
Smart grid (Y04S)	1,291	2,421	146	4	377	709	16	2
Y02 or Y04 patent score (1-3)	**2**	**3**	**2**	**1**	**2**	**2**	**1**	**1**
Percentage of total patents (%)	2.33	2.6	2.17	1.25	2.29	3.78	2.5	1.16
Total percentage score (1-3)	2	3	2	1	2	3	2	1
Average category score	**2**	**3**	**2**	**1**	**2**	**2.5**	**1.5**	**1**

[a] For countries in which total patents exceeds the output limit of the database, the total is estimated from the total of sub-codes

Table A9. Sub-indicators for benchmarking national patents in clean technologies (II)

National patents in clean technologies	IT	LV	NZ	PL	PT	RO	TN	ZA
Total Y02 or Y04 patents [a]	10,712	178	3,245	3,313	2,607	1,391	0	5,639
Building technologies (Y02B)	1,595	8	528	411	406	127	0	670
Energy generation (Y02E)	4,896	155	2,032	2,048	1,674	930	0	3,244
Transportation (Y02T)	3,926	13	387	686	395	283	0	1,167
Capture and storage (Y02C)	146	1	119	113	76	31	0	408
Smart grid (Y04S)	149	1	179	55	56	20	0	150
Y02 or Y04 patent score (1-3)	**2**	**1**	**1**	**2**	**2**	**1**	**0**	**1**
Percentage of total patents (%)	**2.23**	2.96	4.87	1.68	2.12	1.97	0	5.42
Total percentage score (1-3)	2	3	3	1	2	1	**0**	3
Average category score	**2**	**2**	**2**	**1.5**	**2**	1	**0**	**2**

[a] For countries in which total patents exceeds the output limit of the database, the total is estimated from the total of sub-codes

Table A10. Number of public, private, and Scimago ranked universities

Universities and research institutes in the local innovation system	Aalborg	Birmingham	Bologna	Cape Town	Christchurch	Constanța	Dublin	Funchal	Gdynia	Glasgow	Hamburg	Johannesburg	Murcia	Reykjavik	Riga	Sfax	Sydney	Tallinn
Number of universities	2	3	1	3	2	1	6	1	1	4	4	3	2	2	2	1	7	2
Public/polytechnic	2	3	1	3	2	1	5	1	1	4	4	3	1	1	1	1	7	2
Private universities/colleges	0	0	0	0	0	0	1	0	0	0	0	0	1	1	1	0	0	0
Scimago Ranked [a]	✓	✓	✓	✓	✓	✓	✓	✓	✓	✓	✓	✓	✓	✓	✓	✓	✓	✓
Located in the city	1	2	1	3	2	1	6	1	1	4	3	3	2	2	2	1	7	2
Located in the country	8	112	65	17	8	21	12	27	56	112	78	17	61	2	3	8	41	4
Concentration in city (%)	12.5	1.8	1.5	17.6	25.0	4.8	50.0	3.7	1.8	3.6	3.8	17.6	3.3	100.0	66.7	12.5	17.1	50.0
University weighted score	3	5	2	6	4	2	12	2	2	8	7	6	4	4	4	2	14	4

[a] Based on Top 1000 higher education institutional rankings in the world [139]; Universities less than 5,000 students are excluded

---

Reference [1] refers to: Ş. Kılkış, Benchmarking the sustainability of urban energy, water and environment systems and envisioning a cross-sectoral scenario for the future, *Renew Sust Energ Rev* 103 (2019) 529–545. http://dx.doi.org/10.1016/j.rser.2018.11.006.

Appendix B. Supplementary material.
This appendix is based on Tables B1 to B4 of the above mentioned data article that is related to the original research article of [1].

Table B1. Processed Data for the Top 25% of the Cities: Pioneering Cities

City	D_1	D_2	D_3	D_4	D_5	D_6	D_7	Index	$\%\Delta M$	Rank
Copenhagen	36.272	50.000	27.069	31.018	37.620	36.985	31.460	36.038	24.1	1
Stockholm	31.151	50.000	31.500	36.104	38.004	39.031	29.563	36.007	24.0	2
Helsinki	36.462	43.333	26.055	39.802	36.486	35.265	27.680	35.348	21.7	3
Espoo	38.568	39.667	20.650	39.802	36.313	36.931	25.807	34.774	19.7	4
Århus	36.839	46.000	26.056	31.920	30.845	36.098	30.746	34.049	17.2	5
Gothenburg	28.007	42.667	29.294	37.323	35.612	39.107	27.023	33.572	15.6	6
Bregenz	40.042	29.667	28.684	28.778	37.709	34.289	23.931	33.494	15.3	7
Klagenfurt	39.852	33.000	26.699	28.128	33.580	36.945	28.867	33.454	15.2	8
Aalborg	32.613	34.000	26.958	31.432	37.095	39.744	28.920	33.378	14.9	9
Reykjavík	36.370	36.333	32.076	29.213	37.491	36.468	17.908	33.333	14.7	10
Vienna	32.677	46.667	28.147	27.690	30.102	37.471	27.502	32.561	12.1	11
Nice	32.874	34.667	24.218	28.382	40.020	31.961	27.136	32.465	11.8	12
Dubrovnik	37.390	29.667	25.084	35.053	37.856	26.139	20.821	31.972	10.1	13
Zagreb	34.569	44.667	24.620	31.191	32.120	25.440	24.893	31.606	8.8	14
Lisbon	33.307	45.667	31.348	32.668	30.807	24.769	21.444	31.587	8.7	15
Karşıyaka	38.514	28.333	28.627	30.879	39.500	15.766	23.736	31.556	8.6	16
Seferihisar	39.549	23.000	29.809	31.242	40.514	19.100	18.077	31.344	7.9	17
Amsterdam	29.422	43.333	20.952	29.396	30.945	37.272	30.281	31.311	7.8	18
Osijek	38.748	34.000	25.272	31.530	35.136	22.253	18.686	31.134	7.2	19
Leuven	32.582	37.333	22.925	25.351	39.048	30.884	20.139	31.114	7.1	20
Riga	38.886	36.000	21.287	37.363	30.208	24.972	20.700	31.025	6.8	21
Barcelona	31.842	46.667	30.480	28.208	29.704	28.056	22.112	30.966	6.6	22

Ohrid	39.492	27.000	18.544	35.905	39.854	26.602	11.170	30.965	6.6	23
Velenje	38.418	33.000	17.150	32.606	36.562	28.190	16.937	30.947	6.5	24
Bucharest [a]	38.125	45.667	23.530	30.959	31.101	18.180	20.217	30.852	6.2	25
Ljubljana	35.587	40.667	18.098	31.976	29.328	33.012	23.159	30.797	6.0	26
Vilnius	38.971	32.667	16.005	35.968	33.922	27.663	17.499	30.704	5.7	27
Bari	36.995	30.667	26.254	28.693	31.950	27.083	25.024	30.687	5.6	28
Pécs	38.027	34.000	20.842	33.509	34.045	25.636	17.091	30.655	5.5	29
Braga	35.997	22.000	26.905	33.153	37.346	27.533	18.586	30.595	5.3	30

[a] District 1 with SEAP

Table B2. Processed Data for the Top 50-75% of the Cities: Transitioning Cities

City	D_1	D_2	D_3	D_4	D_5	D_6	D_7	Index	$\%\Delta M$	Rank
Frankfurt	29.466	44.667	22.026	30.030	26.947	32.649	33.254	30.594	5.3	31
Karlovac	40.278	23.000	22.895	32.624	36.907	23.663	17.885	30.591	5.3	32
Maribor	36.367	39.667	17.177	30.889	34.526	30.285	14.127	30.496	5.0	33
Sydney	32.478	38.333	22.931	32.548	23.532	35.251	33.009	30.475	4.9	34
Naples	35.798	35.000	21.635	28.662	31.754	29.034	24.230	30.461	4.9	35
Valencia	36.350	34.667	29.707	29.947	29.179	29.105	18.588	30.401	4.7	36
Venice	35.520	31.667	17.709	28.218	36.757	29.167	21.643	30.380	4.6	37
Rijeka	40.086	29.667	18.125	33.950	34.687	21.492	19.178	30.316	4.4	38
Izola	38.136	23.667	18.205	32.780	36.672	30.767	16.849	30.289	4.3	39
Vila Nova de Gaia	34.145	20.667	28.500	31.723	36.921	26.014	21.840	30.138	3.7	40
Grenoble	32.657	32.667	22.490	28.624	30.982	30.920	28.779	30.110	3.6	41
Grand Lyon	25.250	44.667	25.402	28.948	32.106	30.815	26.160	30.030	3.4	42
Glasgow	30.897	39.333	17.065	32.803	27.605	35.228	28.594	30.012	3.3	43
Celje	38.337	25.333	19.228	31.232	35.835	26.386	17.206	29.834	2.7	44

Turin	36.401	46.667	17.477	26.355	26.198	27.978	23.913	29.799	2.6	45
Kranj	36.539	24.000	17.717	32.019	38.443	27.366	16.492	29.797	2.6	46
Milan	32.637	40.000	18.546	26.802	33.59	26.225	23.476	29.752	2.4	47
Zadar	39.186	26.333	19.841	34.724	34.242	20.656	18.107	29.709	2.3	48
Málaga	35.617	32.667	24.450	28.486	33.128	25.813	17.516	29.663	2.1	49
Dublin	31.803	32.667	19.350	33.515	29.765	35.224	22.816	29.656	2.1	50
Pula	36.775	29.667	21.631	34.645	33.480	20.803	18.464	29.597	1.9	51
Bologna	34.404	34.000	16.449	28.786	34.524	27.487	20.619	29.516	1.6	52
Bornova	37.633	31.333	28.543	26.902	32.619	15.766	21.514	29.478	1.5	53
Porto	34.572	20.667	27.932	31.122	30.823	27.533	26.285	29.422	1.3	54
Budapest	30.624	46.667	18.776	33.752	29.088	23.629	22.059	29.380	1.1	55
Zaragoza	34.428	42.667	23.139	30.073	27.486	25.823	17.739	29.304	0.9	56
Pisa	34.460	27.333	26.852	28.839	34.189	21.840	21.063	29.299	0.9	57
Genoa	35.362	41.667	22.180	29.083	25.823	26.193	21.286	29.259	0.7	58
Heraklion	36.924	26.333	25.018	29.587	32.785	25.184	15.896	29.127	0.3	59
Patras	36.954	29.667	22.863	26.764	34.032	26.851	12.873	29.078	0.1	60

Table B3. Processed Data for the Lower 25-50% of the Cities: Solution-Seeking Cities

City	D_1	D_2	D_3	D_4	D_5	D_6	D_7	Index	%ΔM	Rank
Bilbao	36.914	26.000	18.356	29.659	31.687	28.936	20.318	29.021	-0.1	61
Funchal	36.557	18.667	21.884	31.842	35.983	24.170	18.452	28.976	-0.3	62
Murcia	35.766	26.333	25.214	28.605	29.840	29.096	18.231	28.814	-0.8	63
Birmingham	29.474	41.667	14.588	33.502	24.589	33.381	27.967	28.810	-0.8	64
Constanța	39.244	23.000	23.328	29.976	35.238	20.370	11.471	28.674	-1.3	65
Varna	35.790	32.000	18.552	28.875	33.581	22.875	16.175	28.652	-1.4	66
Kalamariá	38.228	26.333	19.254	29.820	32.766	23.517	15.547	28.593	-1.6	67
Nitra	37.690	26.333	21.432	29.220	34.135	23.318	12.517	28.589	-1.6	68
Sevilla	34.520	29.333	28.344	28.153	28.131	25.349	20.231	28.584	-1.6	69
Volos	36.520	28.667	26.901	28.052	31.078	23.517	13.761	28.536	-1.8	70
Burgas	33.879	33.000	20.061	31.353	32.954	22.318	15.103	28.444	-2.1	71
Florence	34.788	26.000	22.38	28.606	30.756	25.826	20.976	28.384	-2.3	72
Paris	21.157	46.667	19.728	28.452	27.128	30.079	33.699	28.283	-2.6	73
Gdynia	36.473	22.000	18.914	32.896	29.942	29.222	16.905	28.169	-3.0	74
Cluj-Napoca	39.584	28.333	20.021	31.511	29.746	20.442	13.257	28.140	-3.1	75
Brașov	40.368	26.333	17.673	32.016	28.378	22.824	14.852	28.035	-3.5	76
Niš	37.294	26.333	21.490	33.406	32.113	13.625	17.257	27.975	-3.7	77
Tallinn	32.552	40.667	13.521	36.587	26.208	27.211	14.758	27.854	-4.1	78
Madrid	27.902	38.000	26.115	29.786	24.700	29.267	21.445	27.759	-4.4	79
Antwerp	29.779	32.667	21.506	25.131	29.141	31.201	21.123	27.739	-4.5	80
Timișoara	38.093	29.333	16.814	31.421	30.387	20.292	12.543	27.591	-5.0	81
Bratislava	32.392	36.000	21.795	29.104	27.391	25.460	14.795	27.463	-5.5	82
Bursa Nilüfer	37.806	20.667	21.412	27.755	31.801	18.997	17.902	27.432	-5.6	83
Thessaloniki	35.515	36.333	19.448	29.820	24.576	25.184	14.658	27.374	-5.8	84
Rome	24.958	34.667	23.796	28.147	28.487	28.313	24.547	27.349	-5.9	85

Tirana	41.220	19.667	24.959	30.779	32.511	11.492	10.314	27.326	-5.9	86
Hamburg	22.602	44.667	20.819	30.903	19.764	31.495	32.992	27.243	-6.2	87
Eskişehir Tepebaşı	37.341	23.667	22.822	28.978	29.924	14.449	18.569	27.105	-6.7	88
Podgorica	38.072	25.333	18.538	34.051	32.422	13.986	9.325	27.025	-7.0	89
Bydgoszcz	34.994	24.000	15.364	29.869	27.548	29.312	17.707	26.897	-7.4	90

Table B4. Processed Data for the Lower 25% of the Cities: Challenged Cities

City	D_1	D_2	D_3	D_4	D_5	D_6	D_7	Index	$\%\Delta M$	Rank
Christchurch	24.386	29.000	20.947	33.188	31.096	31.286	16.698	26.873	-7.5	91
Sofia	30.995	36.000	19.847	30.115	25.619	20.933	19.794	26.701	-8.1	92
Cologne	21.375	44.667	21.600	30.376	19.043	31.678	31.167	26.649	-8.3	93
Antalya	33.505	30.333	24.151	26.206	26.422	17.294	18.212	26.301	-9.5	94
Salé	40.571	20.667	20.140	30.476	29.029	8.468	14.902	26.129	-10.1	95
Skopje	37.806	33.000	17.995	19.149	27.305	19.747	13.622	26.089	-10.2	96
Bijeljina	35.052	22.000	20.071	32.380	34.585	10.566	9.074	26.021	-10.4	97
Ostrava	37.366	28.333	18.755	19.597	25.611	26.193	14.159	26.003	-10.5	98
Rio de Janeiro	30.399	17.167	34.494	37.420	18.455	19.169	27.465	25.981	-10.6	99
Berlin	20.842	46.667	18.406	30.426	11.153	35.121	37.871	25.769	-11.3	100
London	14.459	46.667	17.605	31.644	15.858	35.874	37.754	25.477	-12.3	101
Incheon	24.992	42.667	15.909	21.172	18.431	30.587	31.346	25.388	-12.6	102
São Paulo	25.543	17.167	32.799	39.594	20.700	19.151	26.393	25.290	-12.9	103
Warsaw	29.522	40.000	16.319	30.137	15.873	28.369	20.477	25.165	-13.4	104
Belgrade	31.023	23.333	18.938	32.402	24.741	16.539	21.677	24.996	-14.0	105
Sfax	39.483	18.667	21.351	21.635	32.081	13.191	5.342	24.960	-14.1	106
Sarajevo	33.194	28.333	15.393	28.008	30.642	12.232	9.129	24.617	-15.3	107

Lviv	34.609	29.333	11.716	24.599	27.207	15.191	14.841	24.470	-15.8	108
Batna	37.271	18.667	16.921	17.890	31.480	10.69	10.6	23.722	-18.3	109
Zenica	36.288	22.000	16.561	24.095	28.615	9.047	6.272	23.219	-20.1	110
Washington D.C.	18.687	29.333	17.579	21.493	20.417	29.437	30.159	22.870	-21.3	111
Bangalore	29.627	18.500	15.692	26.179	27.054	9.159	22.037	22.839	-21.4	112
Bogotá	27.452	26.333	15.786	27.393	21.008	19.157	12.93	22.112	-23.9	113
Nagoya	16.759	38.000	18.840	25.605	7.176	26.927	32.134	20.999	-27.7	114
Istanbul [a]	25.372	11.667	21.687	25.133	18.673	13.269	24.609	20.544	-29.3	115
Athens	21.495	18.667	27.139	25.932	11.269	23.579	20.158	20.125	-30.7	116
Johannesburg	20.641	13.667	16.734	19.993	13.221	18.972	19.935	17.479	-39.8	117
Cape Town	19.586	14.667	23.539	26.691	6.476	21.79	18.009	17.446	-39.9	118
Beijing	16.532	32.500	16.522	22.140	1.903	18.253	30.695	17.433	-40.0	119
Tianjin	13.698	21.833	14.767	23.521	1.667	16.09	30.695	15.277	-47.4	120

[a] 12 districts on the Thrace side

Reference [1] refers to: Ş. Kılkış, Benchmarking the sustainability of urban energy, water and environment systems and envisioning a cross-sectoral scenario for the future, *Renew Sust Energ Rev* 103 (2019) 529–545. http://dx.doi.org/10.1016/j.rser.2018.11.006.

Appendix C. Supplementary material.

This appendix contains additional references after reference [11] in the reference list of the above-mentioned data article, which is related to the original research article of reference [1].

[12] Aalborg Kommune, Energy Strategy for Aalborg Municipality until 2030 (Energistrategi for Aalborg Kommune frem til 2030), 2011.

[13] City of Aalborg, Climate Strategy 2012-2015 Mitigation, 2011.

[14] Aalborg Kommune, Municipality Climate Report (Klimakommune Rapport), 2015.

[15] SparEnergi, Energy and CO_2 Accounts Aalborg Municipality 2015 (Energi- og CO_2-regnskabet Aalborg Kommune 2015). http://sparenergi.dk/offentlig/vaerktoejer/energi-og-co2-regnskab/aalborg?year=2015.

[16] Birmingham City Council, Birmingham Sustainable Energy Action Plan 2005 -2020, 2011.

[17] Birmingham Monitoring Report 2017. https://www.covenantofmayors.eu/about/covenant-community/signatories/progress.html?scity_id=1902.

[18] Comune di Bologna, Bologna Action Plan for Sustainable Energy (Bologna Piano D'Azione per L'Energia Sostenibile), 2012.

[19] Bologna Monitoring Report 2015. https://www.covenantofmayors.eu/about/covenant-community/signatories/progress.html?scity_id=1965.

[20] Sustainable Energy Africa, State of Energy in South African Cities, 2015.

[21] City of Cape Town Energy, Environment and Spatial Planning Directorate, Cape Town Energy and Climate Change, 2006.

[22] City of Cape Town, Cape Town Energy 2040: Towards a more Resilient, Low Carbon and Resource Efficient Future for Cape Town, 2015.

[23] Christchurch City Council, Sustainable Energy Strategy for Christchurch, Sustainable Energy Strategy for Christchurch 2008–18, 2007.

[24] Christchurch City Council, Christchurch Energy Action Plan 2015, 2015.

[25] Municipiului Constanța, Sustainable Energy Action Plan of Constanta (Planul de Acțiune privind Energia Durabilă a municipiului Constanța), 2016.

[26] Dublin City Council, Dublin City Sustainable Energy Action Plan 2010 – 2020, 2010.

[27] Dublin City Council, Dublin City Sustainable Energy Action Plan, Monitoring and Progress Report, 2014.

[28] Município do Funchal, Action Plan for Sustainable Energy (Plano de Acção para a Energia Sustentável), 2012.

[29] Região Autónoma da Madeira, Sustainable Energy Action Plan of Madeira Island, 2012.

[30] Município do Funchal, Sustainable Energy Action Plan, 1st Implementation Report (Plano de Acção para a Energia Sustentável, 1º Relatório de Implementação), 2015.

[31] City of Gdynia, Action Plan for Sustainable Energy for Gdynia by 2020 (Plan dzialan na rzecz zrownowazonej energii dla Gdyni do roku 2020), 2012.

[32] Gdynia Monitoring Report 2017. https://www.covenantofmayors.eu/about/covenant-community/signatories/progress.html?scity_id=3949.

[33] Glasgow City Council, Sustainable Glasgow: Energy and Carbon Masterplan, 2011.

[34] Glasgow Monitoring Report 2015. https://www.covenantofmayors.eu/about/covenant-community/signatories/progress.html?scity_id=2434.

[35] The Glasgow Indicators Project. Energy Consumption. http://www.understandingglasgow.com/indicators/environment/energy_consumption.

[36] Hamburg Ministry of Environment and Energy, Hamburg – European Green Capital: 5 Years On, 2016.

[37] Statistical Office for Hamburg and Schleswig-Holstein. Energy Balance and CO_2 Balance for Hamburg (Energiebilanz und CO_2-Bilanzen für Hamburg 2015), 2017.

[38] EU Transform Project, City Transformation Agenda Hamburg, 2015.
[39] Ayuntamiento de Murcia, Murcia Sustainable Energy Action Plan (Plan de Acción de Energía Sostenible de Murcia), 2010.
[40] Murcia Monitoring Report 2017. https://www.covenantofmayors.eu/about/covenant-community/signatories/progress.html?scity_id=1709.
[41] City of Reykjavík, Baseline Emission Inventory and Sustainable Energy Action Plan, 2011.
[42] City of Reykjavík, City of Reykjavík's Climate Policy: Goals for carbon-neutrality and climate change adaptation along with an action plan until 2020, 2016.
[43] Reykjavík Monitoring Report 2015. https://www.covenantofmayors.eu/about/covenant-community/signatories/progress.html?scity_id=3309.
[44] Orkustofnun, OS-2017-T010-01: Final Heat Use in Iceland 2016 by District Heating Area, 2017.
[45] Rigas Energetikas Agentura, Riga Smart City Sustainable Energy Action Plan 2014-2020, 2014.
[46] Tunisie Commune de Sfax, Action Plan for Sustainable Energy (Plan d'action en faveur de l'énergie durable), 2016.
[47] City of Sydney, Environmental Action 2016 – 2021 Strategy and Action Plan, 2017.
[48] City of Sydney, Decentralised Energy Master Plan Renewable Energy 2012–2030, 2013.
[49] City of Sydney, Transport Strategies Actions: Connecting Our Cities, 2017.
[50] C40 Cities, Sydney Emissions, 2017. http://www.c40.org/cities/sydney#city-emissions.
[51] City of Tallinn, Energy Efficiency Action Plan for Tallinn, 2011.
[52] Tallinn Monitoring Report 2015. https://www.covenantofmayors.eu/about/covenant-community/signatories/progress.html?scity_id=1949.
[53] Australian Government Department of Environment and Energy, State and Territory Greenhouse Gas Inventories 2015, 2017.
[54] Covenant of Mayors for Climate and Energy. http://www.covenantofmayors.eu/.
[55] Cities Climate Leadership Group, The Power of C40 Cities, 2018. http://www.c40.org/cities.
[56] JRC-CoM, The Covenant of Mayors for Climate and Energy Reporting Guidelines, 2016.
[57] CoM, Plans and Actions. https://www.covenantofmayors.eu/plans-and-actions/action-plans.html.
[58] C40 City Adviser for Athens, Reducing Greenhouse Gas Emissions in the City of Athens 2017. https://resilientathens.files.wordpress.com/2017/06/cap-part-a-mitigation-plan.pdf.
[59] Secretariat for Administration – Sector for Statistics, Statistical Yearbook of Belgrade, 2015.
[60] Republic of Macedonia State Statistical Office, Energy consumption in households – Total energy consumed per household and per capita per region, 2015.
[61] Zagreb Monitoring Report 2015. https://www.covenantofmayors.eu/about/covenant-community/signatories/progress.html?scity_id=2019.
[62] Budapest Municipality City Administration Department, Budapest Environmental State Evaluation 2015. http://budapest.hu/Documents/BpKAE_2015_honlapra.pdf.
[63] Barcelona Monitoring Report 2017. http://www.covenantofmayors.eu/about/covenant-community/signatories/progress.html?scity_id=1950.
[64] Málaga Monitoring Report 2015. https://www.covenantofmayors.eu/about/covenant-community/signatories/progress.html?scity_id=1699.
[65] Rijeka Monitoring Report 2016. https://www.covenantofmayors.eu/about/covenant-community/signatories/progress.html?scity_id=1815.
[66] University of Zagreb FSB, Sustainable Energy and Climate Action Plan of Dubrovnik, 2017.
[67] World Bank, Mitigation Action Assessment Protocol to the Sustainable Energy and Climate Action Plan of Dubrovnik, 2017.
[68] SparEnergi, Energy and CO_2 Accounts Aarhus Municipality 2015.
[69] City of Aarhus, Climate Plan 2016–2020, 2016.
[70] City of Aarhus, On the Way to Fossil Freedom: A Climate Strategy for Aarhus. http://reader.livedition.dk/aarhuskommune/748/.
[71] Euroheat and Power, Power plant uses heat from the sea to warm up houses in Aarhus, 2017.

[72] Secretaría Distrital de Ambiente, Inventory of Greenhouse Gas Emissions for Bogotá D.C. Base line (Inventario de Emisiones de Gases Efecto Invernadero de Bogotá D.C. Línea Base), 2014.
[73] Länderarbeitskreis Energiebilanzen. Energiebilanz und CO_2-Bilanz in NRW. http://www.lak-energiebilanzen.de/laenderbilanzen/.
[74] Eskişehir Tepebaşı Monitoring Report 2017. https://www.covenantofmayors.eu/about/covenant-community/signatories/progress.html?scity_id=6730.
[75] Helsinki Region Environmental Services, Greenhouse gas emissions from the metropolitan area (Pääkaupunkiseudun kasvihuonekaasupäästöt), 2017. https://hsy.fi/fi/asiantuntijalle/ilmastonmuutos/hillinta/seuranta/Sivut/Paastot.aspx.
[76] Statistik Hessen. Energiebilanz und CO_2-Bilanz. http://www.statistik-hessen.de/publikationen/download/277/index.html.
[77] Câmara Municipal de Lisboa, Lisboa Monitoring Report 2017. https://www.covenantofmayors.eu/about/covenant-community/signatories/progress.html?scity_id=1872.
[78] Municipality of Milan, Sustainable Energy Action Plan (Piano di Azione per l'Energia Sostenibile). http://www.comune.milano.it/wps/portal/ist/it/servizi/ambiente/Energia/PAES.
[79] Milan Monitoring Report 2017. https://www.covenantofmayors.eu/about/covenant-community/signatories/progress.html?scity_id=1834.
[80] Nagoya City Environment Bureau Environment Planning Section. Greenhouse Gas Survey Results. http://www.city.nagoya.jp/kankyo/cmsfiles/contents/0000076/76977/gaiyou2013.pdf.
[81] Paris City Hall, Blue Climate Energy Annex to the 2017 BP proposed to the Paris Council (Bleu Climat Énergie), 2016.
[82] Observatory of Territorial Climate-Energy Plans, Paris GPC Reporting Tool Official Summary. https://www.territoires-climat.ademe.fr/opendata.
[83] City of Paris, Assessment of Paris Climate and Energy Action Plan 2004-2014, 2016.
[84] Municipality of Pisa, Consumo energetico ed Emissioni di CO_2 2015. http://www.ambiente.comune.pisa.it/StatPortal/CatalogoDati/TemplateInstancePrinter.ashx?UID=36af9fb3-7cb9-42ff-80d0-1e22907f8e4f.
[85] Stockholm City Executive Office, Strategy for a fossil-fuel free Stockholm by 2040, 2016.
[86] Stockholm City, Environment Program 2016-2019. http://www.stockholm.se/miljoprogrammet.
[87] Stockholm City, City's Environmental Database Miljöbarometern. http://miljobarometern.stockholm.se/.
[88] Stockholm Monitoring Report 2015. http://www.covenantofmayors.eu/about/covenant-community/signatories/progress.html?scity_id=1888.
[89] City of Vienna, Energy Report of the City of Vienna (Energiebericht der Stadt Wien), 2016.
[90] City of Warsaw, Warsaw Sustainable Energy Action Plan Monitoring Emission Inventory 2014, 2017.
[91] C40 Cities, Washington DC Climate Data. https://www.globalcovenantofmayors.org/cities/washington-dc/.
[92] Environmental Protection Agency, Energy-Related Carbon Dioxide Emissions by State 2000-2014, 2017. https://www.eia.gov/environment/emissions/state/analysis/.
[93] Zaragoza City Hall, Consumption of Energy and CO_2 Emissions in Zaragoza. https://www.zaragoza.es/ciudad/medioambiente/energia/consumoenergia.htm#consumopaes.
[94] Zadar Monitoring Report 2016. https://www.covenantofmayors.eu/about/covenant-community/signatories/progress.html?scity_id=5710.
[95] WHO Ambient (Outdoor) Air Pollution in Cities Database 2016, 2017. http://www.who.int/phe/health_topics/outdoorair/databases/cities/en/.
[96] Urban Waste Water Treatment Directive Site for Europe, 2017. http://uwwtd.oieau.fr/.

[97] European Environment Agency, Urban Waste Water Treatment Map, 2018. https://eea.maps.arcgis.com/apps/MapJournal/index.html?appid=7fa4f0267d8249888b077803714e39fe&embed=true.
[98] NASA Langley Research Center, POWER Data Access Viewer Metrology and Solar Data, NASA Earth Science/Applied Science Program. https://power.larc.nasa.gov/data-access-viewer/.
[99] Enerdata, Energy Efficiency Indicators: Ratio Final/Primary Intensity, 2016.
[100] JRC Photovoltaic Geographical Information System (PVGIS) Incident Global Irradiation for the Chosen Location, 2017. http://re.jrc.ec.europa.eu/pvg_tools/en/tools.html.
[101] IRENA/DTU, Global Atlas for Renewable Energy DTU Global Wind Atlas Wind Speed Maps. https://irena.masdar.ac.ae/gallery/#tool/10.
[102] GENI Global Energy Research Institute, Atlas of Geothermal Resources in Europe Heat-flow Density. http://www.geni.org/globalenergy/library/renewable-energy-resources/world/europe/geo-europe/index.shtml.
[103] Enerdata, Global Energy Statistical Yearbook, 2017.
[104] IEA Statistics by Country Balances and Electricity and Heat Data Tables, 2017. https://www.iea.org/statistics.
[105] Department of Environmental Affairs South Africa. Air Quality. https://www.environment.gov.za/sites/default/files/reports/environmentoutlook_chapter10.pdf.
[106] Agência Portuguesa do Ambiente. QualAr Base de Dados Online sobre a Qualidade de Ar. https://qualar.apambiente.pt/qualar/index.php.
[107] A. Calcott, J. Bull, Ecological footprint of British city residents, WWF. http://assets.wwf.org.uk/downloads/city_footprint2.pdf.
[108] WFN, Water Footprint Statistics. http://waterfootprint.org/en/resources/water-footprint-statistics/.
[109] A. Hoekstra, M. Mekonnen, The Water Footprint of Humanity, Proceedings of the National Academy of Sciences 109(9) (2012), 3232–3237.
[110] G. Carr, C. Rickwood, Water Quality: Development of an Index to assess Country Performance, UNEP GEMS/Water Programme, 2008.
[111] T. Srebotnjak, G. Carr, A. Sherbinin, C. Rickwood, A Global Water Quality Index and Hot-Deck Imputation of Missing Data, Ecol Indic 17 (2012) 108–119.
[112] GFN, Global Footprint Network National Footprint Accounts (2017 Edition). http://www.footprintnetwork.org.
[113] W. Baabou, N. Grunewald, C. Ouellet-Plamondon, M. Gressot, A. Galli, The Ecological Footprint of Mediterranean Cities: Awareness Creation and Policy Implications, Envir Sci Policy 69 (2017) 94–104.
[114] University of Canberra. Urban Competitiveness Index: Measuring the Competitiveness of Australian Cities. http://www.canberra.edu.au/media-centre/attachments/pdf_folder/Measuring-the-competitiveness-of-Australian-Cities.pdf.
[115] WHO, Air quality guidelines for particulate matter, ozone, nitrogen dioxide and sulfur dioxide (Global update 2005), Geneva, 2006.
[116] ACI Airport Carbon Accreditation, Accredited Airports Around the World, 2017. http://www.airportcarbonaccreditation.org/airport/participants.html.
[117] Ecorys, Study on European energy intensive industries – The usefulness of estimating sectoral price elasticities, Methodological Review 2009; ENTR/06/054, 1–13.
[118] UWWTD-REP, Terms and Definitions of the Urban Waste Water Treatment Directive 91/271/EEC, 2007.
[119] A. Kaczorowska, J. Kain, J. Kronenberg, D. Haase, Ecosystem services in urban land use planning: Integration challenges in complex urban settings - Case of Stockholm, Ecosyst Serv 22 (2016) 204–212.
[120] European Climate Adaptation Platform. https://climate-adapt.eea.europa.eu/tools/urban-adaptation/.

[121] Eurostat, Municipal Waste by NUTS 2 Regions – Pilot Project Data, 2016 (Extracted on 12 August 2017).
[122] European Commission, Assessment of separate collection schemes in the 28 capitals of the EU, 2015.
[123] World Bank, What a Waste Global Database. https://datacatalog.worldbank.org/dataset/what-waste-global-database.
[124] Food and Agriculture Organization, Aquastat Municipal Wastewater Production, Collection, Treatment and Use Database, 2016. http://www.fao.org/nr/water/aquastat/wastewater/index.stm#db.
[125] Department of Water and Sanitation, Green Drop Assessment Analysis: Comparative Performance, 2017.
[126] OECD Metropolitan Areas Database. http://www.oecd.org/gov/regional/measuringurban.
[127] EEA Percentage of Green Urban Areas in EU-27 Core Cities. https://www.eea.europa.eu/data-and-maps/figures/percentage-of-green-urban-areas-1.
[128] IUCN World Database on Protected Areas, 2018. http://www.protectedplanet.net/.
[129] Ramsar Sites Information Service. https://rsis.ramsar.org/.
[130] World Bank GDP per Capita PPP International Dollars, 2017. http://data.worldbank.org/indicator/NY.GDP.PCAP.PP.CD.
[131] Gallup, Behavioral Economics of GDP Growth and Global Wellbeing. Washington D. C, 2010.
[132] Eurostat, Education and Training Database. http://ec.europa.eu/eurostat/web/education-and-training/data/database.
[133] OECD, Education Policy Outlook Country Profiles. http://www.oecd.org/edu/profiles.htm.
[134] R. Barro, J. Lee, A New Data Set of Educational Attainment in the World. http://www.barrolee.com/.
[135] EPO, Finding Sustainable Technologies in Patents, Munich, 2013.
[136] JRC Research and Innovation Observatory (RIO). https://rio.jrc.ec.europa.eu/en.
[137] OECD/UNESCO, Main Science, Technology and Innovation Statistics Database (June 2017 Release), 2017.
[138] EPO European Patent Office Espacenet. http://www.epo.org/searching/free/espacenet.html.
[139] Scimago Institutions Rankings. http://scimagoir.com/rankings.php.
[140] Scimago Research Group Journal & Country Rank. http://www.scimagojr.com/countrysearch.php.
[141] Ş. Kılkış, Benchmarking the Sustainability of Urban Energy, Water and Environment Systems with the SDEWES City Index and Envisioning Scenarios for the Future, Plenary Lecture 12th SDEWES Conference Dubrovnik, October 4–8 2017. http://www.dubrovnik2017.sdewes.org/lectures.php.
[142] DBDH, Aalborg District Heating. https://dbdh.dk/members/aalborg-forsyning/.
[143] ENGIE, Birmingham District Energy Scheme. https://www.engie.co.uk/energy/district-energy/birmingham/.
[144] Eskom, Ankerlig Open Cycle Gas Turbine Power Plant. http://www.eskom.co.za/Whatweredoing/ElectricityGeneration/PowerStations/Pages/Ankerlig.aspx.
[145] CET Constanta CHPP Power Plant Romania, Global Energy Observatory. http://globalenergyobservatory.org/geoid/41137.
[146] D. Gartland, Dublin City Spatial Energy Demand Analysis, Codema-Dublin City Council, 2015.
[147] Electricidade da Madeira, Centrais termoelétricas. https://www.eem.pt/pt/conteudo/sistema-elétrico/produção/centrais-termoelétricas/.
[148] OPEC Sp. Wejherowo. https://opecgdy.com.pl/dla-klienta/podlacz-sie-do-sieci/rozwijamy-sie-w-kazdym-kierunku/wejherowo/.
[149] J. Gerbitz, K. Müller, K. Jacob, Hamburg Wilhelmsburg Implementation Plan, Transform Project, 2014.
[150] Kelvin A and B Thermal Power Station South Africa, Global Energy Observatory. http://globalenergyobservatory.org/form.php?pid=43755.

[151] Secretary General Department of Planning and Studies, Boletín De Estadísticas Energéticas De Cogeneración (7th Edition), 2016.
[152] Utilitas, OÜ Utilitas Tallinna Elektrijaam. http://www.elektrijaam.ee/_old/ettevottest.
[153] European Power to Gas. Power-to-gas Projects in Europe, 2018. http://europeanpowertogas.com/projects-in-europe/.
[154] Aalborg CSP, A world first renewable energy system inaugurated in Denmark. https://www.aalborgcsp.com/news-events/newstitle/news/a-world-first-renewable-energy-system-inaugurated-in-denmark/.
[155] Gruppo Hera, Sustainability Report 2016, 2017. http://eng.gruppohera.it/group/social_responsibility/sustainability_report/.
[156] NZ Geothermal Association, Ground Water for Airport Cooling and Heating. https://www.gns.cri.nz/Home/Learning/Science-Topics/Earth-Energy/Case-Studies.
[157] CES, Kogeneracja Dla Oczyszczalni Ścieków Dębogórze W Gdyni. http://www.kogeneracjaces.pl/realizacja/64/oczyszczalnia-sciekow.html.
[158] R. Pieras, BIOCELL - Energy self-sustaining and environmental footprint reduction on wastewater treatment plants via fuel cells (LIFE07 ENV/E/000847), 2012.
[159] Sydney Central Park district energy scheme tri-generation. https://www.clarke-energy.com/2015/sydney-central-park-district-energy-scheme-reduces-carbon-emissions-using-tri-generation/.
[160] Innovation Alliance of Hamburg - Schleswig-Holstein, North German Energy Transition Hamburg, 2017.
[161] National Plans for Increasing the Number of Nearly Zero-Energy Buildings. https://ec.europa.eu/energy/en/topics/energy-efficiency/buildings/nearly-zero-energy-buildings.
[162] D. D'Agostino, P. Zangheri, B. Cuniberti, D. Paci, P. Bertoldi, Synthesis Report on the National Plans for Nearly Zero Energy Buildings (NZEBs), JRC, Ispra, 2016.
[163] BPIE, Nearly Zero Energy Buildings Definitions Across Europe. http://bpie.eu/uploads/lib/document/attachment/128/BPIE_factsheet_nZEB_definitions_across_Europe.pdf.
[164] Port of Aalborg becomes the first CO_2-neutral port in Denmark, 2017. https://safety4sea.com/port-of-aalborg-becomes-the-first-co2-neutral-port-in-denmark/.
[165] Communities and Local Government, Code for Sustainable Homes Technical Guide, 2010.
[166] PVC Construct, Ready for 2020 The 'nearly zero-energy buildings' of Ozzano dell'Emilia, Italy, 2011.
[167] Green Building Council, Growthpoint's Greenfield Industrial Park achieves SA's first industrial Green Star rating, 2016.
[168] New Zealand's greenest building, 2018. https://ngaitahu.iwi.nz/new-zealands-greenest-building/.
[169] Dublin City Council, Dublin City Development Plan 2016–2011, 2016.
[170] Clyde Waterfront, Glasgow City Centre urban redevelopment. http://www.clydewaterfront.com/projects/glasgow-city-centre.
[171] IBA Hamburg GmbH, Energy Bunker, International Building Exhibition Hamburg, 2014.
[172] City of Sydney, Net-zero apartment buildings, 2018. http://www.cityofsydney.nsw.gov.au/vision/towards-2030/sustainability/carbon-reduction/net-zero-apartment-buildings.
[173] C. Derksema, N. Midlam, Accelerating Net-Zero High-Rise Residential Buildings in Australia, 2016.
[174] Research in Estonia, Estonian buildings need sustainable solutions, 2017. http://researchinestonia.eu/2016/10/28/estonian-buildings-need-sustainable-solutions/.
[175] D. D'Agostino, P. Zangheri, L. Castellazzi, Towards Nearly Zero Energy Buildings in Europe: A Focus on Retrofit in Non-Residential Buildings, Energies 10(1) (2017) 1–15.

[176] Green Building Council, GBCA joins ground breaking project to build a carbon zero future. https://new.gbca.org.au/news/gbca-media-releases/gbca-joins-ground-breaking-project-build-carbon-zero-future/.
[177] Demographia, Demographia World Urban Areas, 13th Annual Edition: 2017:04, 2017.
[178] J. Serradell, J. Kennes, World Metro Database. http://mic-ro.com/metro/table.html.
[179] Servizio Ferroviario Metropolitano Bologna, Bologna Metropolitan Railway Service. http://www.sfmbo.it//Engine/RAServePG.php/P/25251SFM0611/T/English-Version.
[180] Horários do Funchal. Eco Line (Linha Eco). http://www.horariosdofunchal.pt/index.php?option=com_content&task=view&id=48&Itemid=331.
[181] Hamburger Hochbahn AG. Emission-Free Buses (Emissionsfreie Busse). https://www.hochbahn.de/hochbahn/hamburg/de/Home/Naechster_Halt/Ausbau_und_Projekte/emissionsfreie_busse.
[182] Sydney Light Rail. http://sydneylightrail.transport.nsw.gov.au/map.
[183] Municipality of Bologna, Bike ride: Public rental bikes (C'entro in bici: Biciclette pubbliche a noleggio). http://www.comune.bologna.it/trasporti/servizi/2:3026/3295/.

Reference [1] refers to: Ş. Kılkış, Benchmarking the sustainability of urban energy, water and environment systems and envisioning a cross-sectoral scenario for the future, *Renew Sust Energ Rev* 103 (2019) 529–545. http://dx.doi.org/10.1016/j.rser.2018.11.006.
